# Carbon dots in cancer therapy: a novel approach to targeted drug delivery and reduced toxicity in gallbladder cancer

**DOI:** 10.3389/fchem.2026.1899601

**Published:** 2026-09-17

**Authors:** Mahvish Fatima, Warda Parveen, Norah M. Almaneai, Attalla F. El-kott, Mohammed A. AlShehri, Waleed Eltantawy, Nosheen Kanwal, Zaheera Saadia

**Affiliations:** 1 Department of Physics, College of Science, Qassim University, Buraidah, Al-Qassim, Saudi Arabia; 2 College of Energy and Minning Engineering, Shandong University of Science and Technology, Qingdao, China; 3 Department of Chemistry, College of Science, Qassim University, Buraidah, Al-Qassim, Saudi Arabia; 4 Department of Biology, College of Science, King Khalid University, Abha, Saudi Arabia; 5 Department of Zoology, Faculty of Science, Damanhour University, Damanhour, Egypt; 6 Department of Biology, Program of Preparatory Year, Batterjee Medical College, Aseer, Saudi Arabia; 7 Department of Obstetrics and Gynecology, College of Medicine, Qassim University, Buraidah, Al-Qassim, Saudi Arabia

**Keywords:** biocompatibility, cholangiocarcinoma (CCA), gallbladder cancer (GBC), hepatobiliary targeting, nanotoxicology, targeted drug delivery

## Abstract

Biliary tract cancers (BTCs), including cholangiocarcinoma and gallbladder carcinoma, are highly aggressive malignancies associated with poor prognosis, late diagnosis, and limited therapeutic options. Conventional chemotherapy offers only modest survival benefits because of inadequate tumor selectivity, rapid systemic clearance, dose-limiting toxicity, and the emergence of chemoresistance. Carbon quantum dots (CQDs) have emerged as promising nanotheranostic agents owing to their ultrasmall size, tunable photoluminescence, excellent aqueous dispersibility, biocompatibility, and versatile surface chemistry, enabling simultaneous drug delivery, imaging, and phototherapy. This review summarizes the evolution of carbon dots into structurally engineered CQDs and discusses their physicochemical, optical, and biological properties that underpin their biomedical applications. Particular emphasis is placed on recent advances in CQD-based drug delivery, fluorescence bioimaging, photothermal therapy, photodynamic therapy, and multifunctional theranostic platforms relevant to BTC management. Although direct studies on CQDs for BTC remain limited, emerging evidence from hepatobiliary and other gastrointestinal cancers highlights their potential to improve targeted drug delivery, tumor imaging, and therapeutic efficacy. The review further identifies critical challenges to clinical translation, including the lack of BTC-specific preclinical models, insufficient pharmacokinetic and long-term biosafety data, and the need for standardized, reproducible CQD synthesis and functionalization protocols. Finally, future research directions are discussed, emphasizing tumor-targeted CQD engineering, multimodal therapeutic strategies, and integration with precision oncology. Collectively, CQDs represent a promising nanoplatform for the development of targeted, image-guided, and minimally invasive approaches for the diagnosis and treatment of biliary tract cancers.

## 1 Introduction

Biliary tract carcinoma (BTC) comprises a group of highly malignant neoplasms that arise from the biliary epithelium and display marked histological and clinical heterogeneity. Clinically and anatomically, BTC is divided into gallbladder carcinoma (GBC), intrahepatic cholangiocarcinoma (iCCA), perihilar cholangiocarcinoma (pCCA), and distal cholangiocarcinoma (dCCA); pCCA and dCCA are frequently grouped together as extrahepatic cholangiocarcinoma (eCCA). As one of the principal primary liver malignancies, BTC has shown a rising incidence in many regions worldwide and consequently represents an increasing global health burden (Recio-Boiles et al., 2024). Its occurrence varies substantially by geography and population: for example, higher rates have been reported in countries such as Chile, Japan, and the Republic of Korea, and recent data indicate that East Asia records the highest age-standardized incidence of iCCA internationally (Qurashi et al., 2025).

Biliary tract cancers (BTC) comprising cholangiocarcinoma (CCA) and gallbladder carcinoma, have dismal prognoses, partly because most cases are diagnosed at advanced stages. For example, 5-year survival for advanced cholangiocarcinoma is <10%, and even resectable cases do poorly (Dana et al., 2025). Conventional chemotherapies (5-fluorouracil, gemcitabine, doxorubicin, cisplatin) are non‐targeted and cause severe side effects and chemoresistance. Given the limited efficacy and substantial systemic toxicity associated with conventional cytotoxic agents, there is an urgent need for new therapeutic directions for cholangiocarcinoma, including chemopreventive and adjuvant approaches. Traditional chemotherapies are largely non-selective and damage healthy tissues alongside tumor cells, producing undesirable adverse effects. Strategies that favor tumor-targeted delivery can increase drug accumulation at the lesion site and promote cellular uptake, thereby improving therapeutic indices. In this context, nanoformulated drug carriers have emerged as a promising route to overcome the limitations of classic chemotherapeutics. In parallel, identifying and exploiting molecular targets that specifically drive tumor growth and progression could enable the design of targeted agents that improve clinical outcomes. These combined approaches offer a rational framework for next-generation drug development against BTC (Sirica, 2005). These limitations demand novel delivery systems for selective transport drug system to BTC tumors, enhancing efficacy while sparing healthy cells. Nanotechnology offers new platforms for precision targeting. In particular, carbon dots (CDs) which are nanoscale, carbon-based particles with bright fluorescence have emerged as promising drug carriers (Sun et al., 2024).

Nanomedicine offers several strategies to meet these goals, and among emerging nanoplatforms, carbon dots (CDs) a family of quasi-spherical, <10 nm, fluorescent carbon nanomaterials have attracted major attention because they combine tunable surface chemistry, (Zavareh et al., 2020), (de Groen et al., 1999) bright photoluminescence for tracking, high aqueous stability, and generally low intrinsic toxicity compared with many inorganic quantum dots. Recent comprehensive reviews and preclinical studies have demonstrated CDs’ potential as theranostic carriers that can both image and deliver therapeutics, making them promising candidates to address the unmet needs of targeted therapy in biliary tract cancers such as GBC (de Groen et al., 1999). Carbon dots are typically synthesized by several approaches such as from small carbon precursors or biomass, and their surface can be readily functionalized with targeting ligands (folic acid, peptides, antibodies), therapeutic cargos (small-molecule drugs, siRNA), or stimuli-responsive groups (pH, redox, photo-responsive moieties). Such modular synthesis permits fine control over size, surface charge and hydrophilicity parameters that govern circulation time, tumor penetration, and cellular uptake (Crista et al., 2020). Importantly, the intrinsic fluorescence of many CDs enables noninvasive tracking of biodistribution, an advantage for preclinical development and potential clinical theranostic use. These synthesis and functionalization capabilities have been summarized across multiple recent surveys of the field, which also highlight advances in green, low-cost routes that improve biocompatibility (Kaurav et al., 2023).

A central advantage of CD-based carriers for cancer therapy is their capability for *active targeting* and *controlled release*. By decorating CDs with moieties that bind tumor-overexpressed receptors (for example, folate for folate-receptor-positive tumors or peptide ligands for amino-acid transporters), researchers have achieved selective uptake into malignant cells while sparing normal tissues. Beyond ligand-based targeting, CDs can be engineered for tumor-responsive release for instance, linking drugs via pH-sensitive bonds that cleave in the acidic tumor microenvironment or utilizing glutathione-sensitive linkers that respond to elevated intracellular reducing conditions (Tringale et al., 2018). Several preclinical studies report superior tumor accumulation and reduced systemic toxicity for drug-loaded CDs compared with free drug or conventional liposomal carriers, and *in vivo* imaging confirms the feasibility of simultaneous tracking and therapy (Li S, 2020). Reviews and recent experimental reports document these multi-modal tactics and demonstrate how metal-doping or heteroatom functionalization improves photophysical properties and therapeutic outcomes (Sun et al., 2024). While the CD literature shows broad efficacy across multiple tumor types, direct preclinical studies focused specifically on gallbladder cancer remain limited. However, evidence from closely related hepatobiliary and gastrointestinal cancer models supports translational plausibility: for example, CD-loaded chemotherapeutics and CD-hybrid nanoparticles have produced substantial tumor inhibition in liver cancer models while exhibiting low toxicity to healthy tissue a proof-of-principle that is highly relevant because GBC shares microenvironmental and vascular characteristics with other hepatobiliary tumors (Llovet and Bruix, 2008). Translating CDs to GBC will require attention to tumor vasculature, desmoplasia, and receptor expression profiles (to select appropriate targeting ligands), but the modularity of CDs makes such tailoring feasible. Additionally, recent high-impact studies have produced tumor-targeted CD platforms with superior delivery of doxorubicin and other chemotherapeutics, demonstrating both enhanced antitumor activity and reduced systemic side effects compared with conventional formulations results that motivate targeted investigations in GBC models (Fawaz et al., 2024). Safety and regulatory considerations are crucial before clinical translation. Although many studies report low cytotoxicity and favorable *in vivo* tolerability for properly synthesized and surface-stabilized CDs, toxicity is highly dependent on precursor materials, surface dopants, size, and dosage. Comprehensive toxicological profiling (acute and chronic), immunogenicity testing, and scaled-up, reproducible manufacturing under Good Manufacturing Practice (GMP) conditions will be essential (Ren et al., 2016). Encouragingly, recent reviews emphasize that with careful design using biocompatible precursors, avoiding heavy-metal dopants, and implementing robust purification CDs can achieve acceptable safety margins for further preclinical development (Bhattacharya et al., 2023). Traditionally, biliary tract cancers (BTCs), including gallbladder carcinoma and cholangiocarcinoma, have been managed with conventional chemotherapeutic drugs such as gemcitabine and cisplatin, which offer limited efficacy and cause significant systemic toxicity. The emergence of carbon-based nanomaterials introduced new therapeutic possibilities, and early studies using carbon dots (CDs) demonstrated improved drug delivery and imaging potential. However, most investigations centered on hepatic or general gastrointestinal cancers, with minimal focus on the biliary system. Moreover, these early CDs lacked structural control, resulting in inconsistent optical and therapeutic behavior.

Recent advances have led to the development of carbon quantum dots (CQDs), a more crystalline and photophysically refined class of carbon nanomaterials, with superior crystallinity, enhanced photovoltaic (charge-transfer) capacity, and tunable photoluminescence extending into the near-infrared region (Karagianni et al., 2023), offering deeper tissue penetration and efficient reactive oxygen species generation for photothermal and photodynamic therapy (Pechnikova et al., 2025). Several comprehensive reviews have summarized the synthesis, physicochemical properties, and biomedical applications of carbon dots across diverse disease models. However, a focused review addressing the application of CDs and carbon quantum dots (CQDs) in biliary tract cancers (BTCs) remains lacking. Unlike previous reviews that broadly discuss carbon nanomaterials in oncology, the present review specifically examines the biological rationale for employing CDs in BTC by integrating recent advances in molecular targeting, tumor-specific drug delivery, fluorescence-guided imaging, photodynamic and photothermal therapy, and pathway-directed nanomedicine. Furthermore, this review critically evaluates the current limitations of preclinical evidence, challenges associated with clinical translation, including pharmacokinetics, biodistribution, large-scale manufacturing, regulatory considerations, and long-term biosafety, and identifies key research priorities necessary for the successful translation of CQD-based nanotherapeutics into clinical management of BTC.

## 2 Clinical burden and therapeutic limitations of biliary tract cancer

### 2.1 Epidemiology of biliary tract cancer

Biliary tract cancer (BTC), which encompasses intrahepatic and extrahepatic cholangiocarcinoma as well as gallbladder carcinoma, is an uncommon but highly lethal group of malignancies whose incidence, mortality, and geographic distribution exhibit marked heterogeneity. Globally, BTC burden has risen over recent decades with important regional hotspots and substantial increases in absolute cases and deaths in some countries (Su et al., 2024). Established etiologic and predisposition factors differ by subtype and region: liver-fluke infection (Opisthorchis/Clonorchis) and hepatolithiasis strongly predispose to cholangiocarcinoma in endemic areas, whereas cholelithiasis, gallbladder polyps, and chronic inflammation are the dominant risks for gallbladder cancer in high-incidence zones such as parts of South Asia and Latin America (Khan et al., 2019).

BTC shows a female predominance for gallbladder primaries and a wide range of age at diagnosis, but most patients present with advanced, often unresectable disease because early symptoms are nonspecific; consequently, population-level 5-year survival remains poor (single-digit to low-teens percent for many stages) (Tirca et al., 2024). Molecular heterogeneity across geographic and etiologic contexts has clinical implications for targeted therapy and trial design, underscoring the need for regionally tailored prevention (e.g., fluke control, gallstone management) and earlier detection strategies to improve outcomes (Søreide et al., 2025). Figure 1 shows the different type of malignancies associated with biliary tract cancer.

**FIGURE 1:**
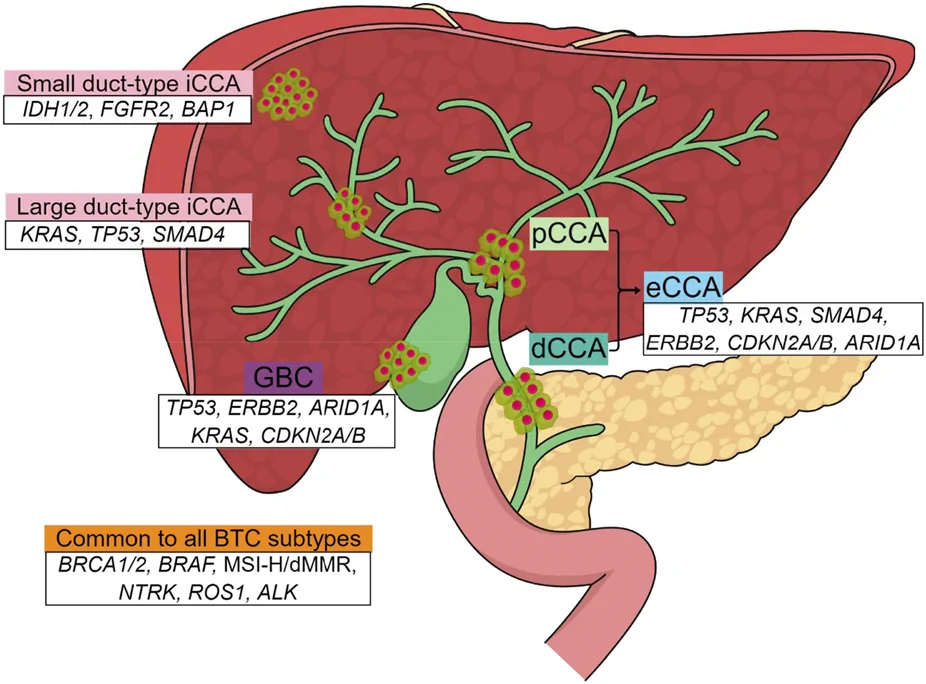
Molecular characteristics of biliary tract cancer according to anatomical location. iCCA, intrahepatic cholangiocarcinoma; pCCA, perihilar cholangiocarcinoma; eCCA, extrahepatic cholangiocarcinoma; dCCA, distal cholangiocarcinoma; GBC, gallbladder cancer. Reprint with permission from (Sang-Hoon Lee), (Recent Advancement in Diagnosis of Biliary Tract Cancer through Pathological and Molecular Classifications); published by (MDPI), (2024) (Roderburg et al., 2024).

### 2.2 Current treatment limitations in biliary tract cancer

Gallbladder cancer (GBC) carries a heavy clinical burden because most patients present with advanced, often unresectable disease, leading to dismal long-term outcomes. The overall population survival is typically in the single digits and even after attempted curative resection recurrence rates remain high (Pandit, 2023). GBC is the most common biliary tract malignancy and is typically diagnosed late because early stages are often asymptomatic or mimic benign biliary disease (e.g., cholelithiasis or cholecystitis). Surgical resection with R0 margins offers the only chance for cure, but a minority of patients (ranging between 10%–35%) are candidates for curative surgery at diagnosis because of late presentation or locally advanced spread. In recent retrospective research, the scientist observed that the patients who were undergone curative resection (R0) for gallbladder cancer showed a 5-year survival rate in the range of 21 %–57%, with variation depending on stage and associated factors. Multivariate analysis identified that higher tumour stage (T status), presence of nodal metastases, and positive surgical margins markedly reduced long-term survival. In contrast, achieving an R0 resection, the absence of lymph node involvement, and lower tumour burden emerged as favourable prognostic factors. The data suggest that only a minority of patients achieve prolonged survival, and that careful patient selection and complete surgical clearance are critical for achieving better outcomes (Balachandran et al., 2006).

For unresectable, locally advanced, or metastatic disease the backbone systemic regimen remains gemcitabine plus cisplatin, based on the randomized ABC-02 trial that established this combination as standard first-line therapy for advanced biliary tract cancers (Valle et al., 2010). Adjuvant therapy options are limited: the phase III BILCAP study showed a survival advantage for adjuvant capecitabine over observation in resected biliary tract cancers and has informed adjuvant practice, but benefit is modest and many patients still relapse (Primrose, 2019). Beyond cytotoxic chemotherapy, targeted and immune therapies have shown promise only in molecularly selected subsets (for example, HER2-amplified tumors or other actionable alterations), but these aberrations occur in a minority of GBCs and broad, validated targeted options are not yet available for most patients; consequently, routine molecular profiling and enrolment in clinical trials are important but access and efficacy remain limiting factors (Zhu et al., 2024). The 2024 systematic review by Zhu et al., comprehensively evaluated the therapeutic landscape of HER2-targeted therapies across multiple cancer types. It highlights that HER2 alterations, amplification, overexpression, or mutation occur not only in breast and gastric cancers but also in lung, biliary tract, colorectal, and other tumors. It also demonstrates that HER2 targeted agents, including monoclonal antibodies, tyrosine kinase inhibitors, and antibody drug conjugates (ADCs), have markedly improved clinical outcomes, particularly in HER2-positive and HER2-low tumors. Some benefits were reported in gastric and biliary tract cancers, where T-DXd achieved response rates above 30% in HER2-positive patients. However, resistance mechanisms such as receptor heterogeneity, downstream pathway activation, and microenvironmental factors, remain key challenges (Zhu et al., 2024). Figure 2 shows the categories of drugs for HERS-2 containing different types of antibodies.

**FIGURE 2:**
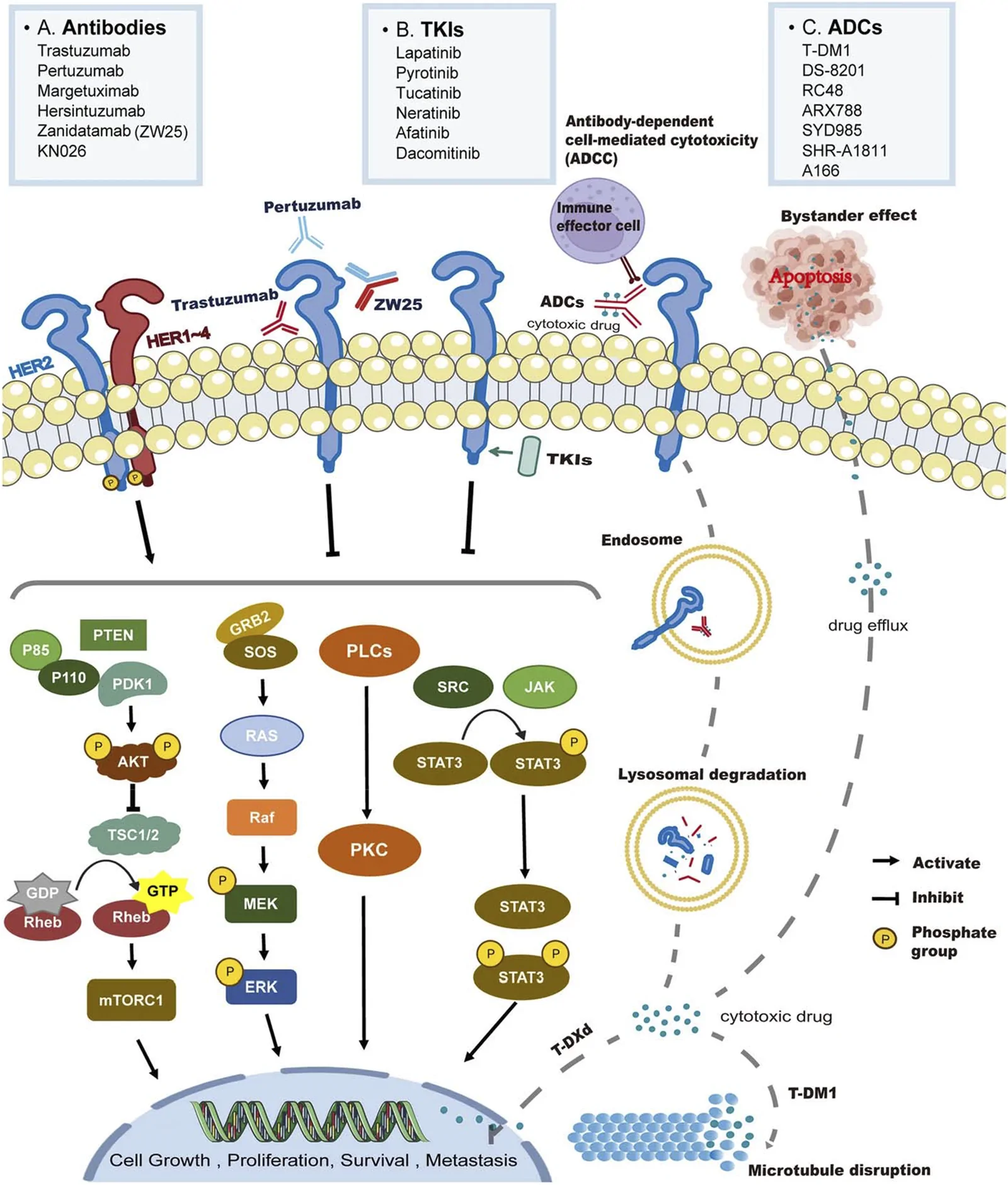
Classification and mechanisms of prevalent anti-HER2-targeting medications. Reprint with permission from (Kunrui Zhu), (HER2-targeted therapies in cancer: a systematic review); published by (Springer Nature), (2024) (Zhu et al., 2024).

These clinical realities highlight an urgent need for therapies that increase tumor selectivity and reduce collateral toxicity for pancreaticobiliary tissues and liver parenchyma surrounding the gallbladder (Zhou et al., 2023).

### 2.3 Why nanomedicine is needed in biliary tract cancer

Biliary tract cancers (BTCs) are a highly heterogeneous group of malignancies that affect both the small intrahepatic and large extrahepatic bile ducts (cholangiocarcinoma) or the gallbladder (gallbladder cancer) (Banales et al., 2020). Biliary tract cancers (BTCs) are refractory to conventional systemic therapy because multiple, overlapping pharmacologic barriers restrict effective intratumoral drug delivery. First, highly desmoplastic stroma and abundant cancer-associated fibroblasts generate dense extracellular matrix and elevated interstitial fluid pressure that physically impede convective transport and reduce microvascular perfusion (Jessel et al., 2025). Second, the tumor vasculature in many BTCs is heterogeneous and poorly perfused, so relying on passive accumulation through the enhanced permeability and retention (EPR) effect yields highly variable and frequently suboptimal drug deposition (Sharifi et al., 2022).

A recent review suggests that Enhanced Permeability and Retention (EPR) effect as a mechanism for nanocarrier accumulation in solid tumors. It also concludes that while the EPR effect remains a key concept in cancer nanomedicine, its clinical reliability is limited by tumor heterogeneity, abnormal vasculature, and elevated interstitial pressure, which restrict uniform nanoparticle delivery. EPR is highly variable across tumor types and patients, and that active transcellular and vesicular transport mechanisms also contribute to nanoparticle uptake beyond passive diffusion. There is need to improve herapeutic efficacy modulation of the tumor microenvironment, engineering of adaptive, stimuli-responsive nanocarriers, and integration of real-time imaging for patient-specific optimization (Sharifi et al., 2022). Third, the hepatobiliary milieu imposes additional pharmacokinetic challenges: biliary excretion, local enzymatic metabolism, and overexpression of drug-efflux transporters (e.g., ABC family pumps) lower intra-tumoral concentrations of many small-molecule cytotoxics while exposing normal tissues to systemic toxicity (Liu et al., 2023). Collectively these factors narrow the therapeutic index of standard agents (e.g., gemcitabine, cisplatin, 5-FU), contribute to primary and acquired chemoresistance, and help explain why objective responses and durable remissions remain uncommon in advanced BTC (Sharifi et al., 2022), (Liu et al., 2023). Figure 3 shows the proposed pathways for the nano-drugs to enter the cancerous area.

**FIGURE 3:**
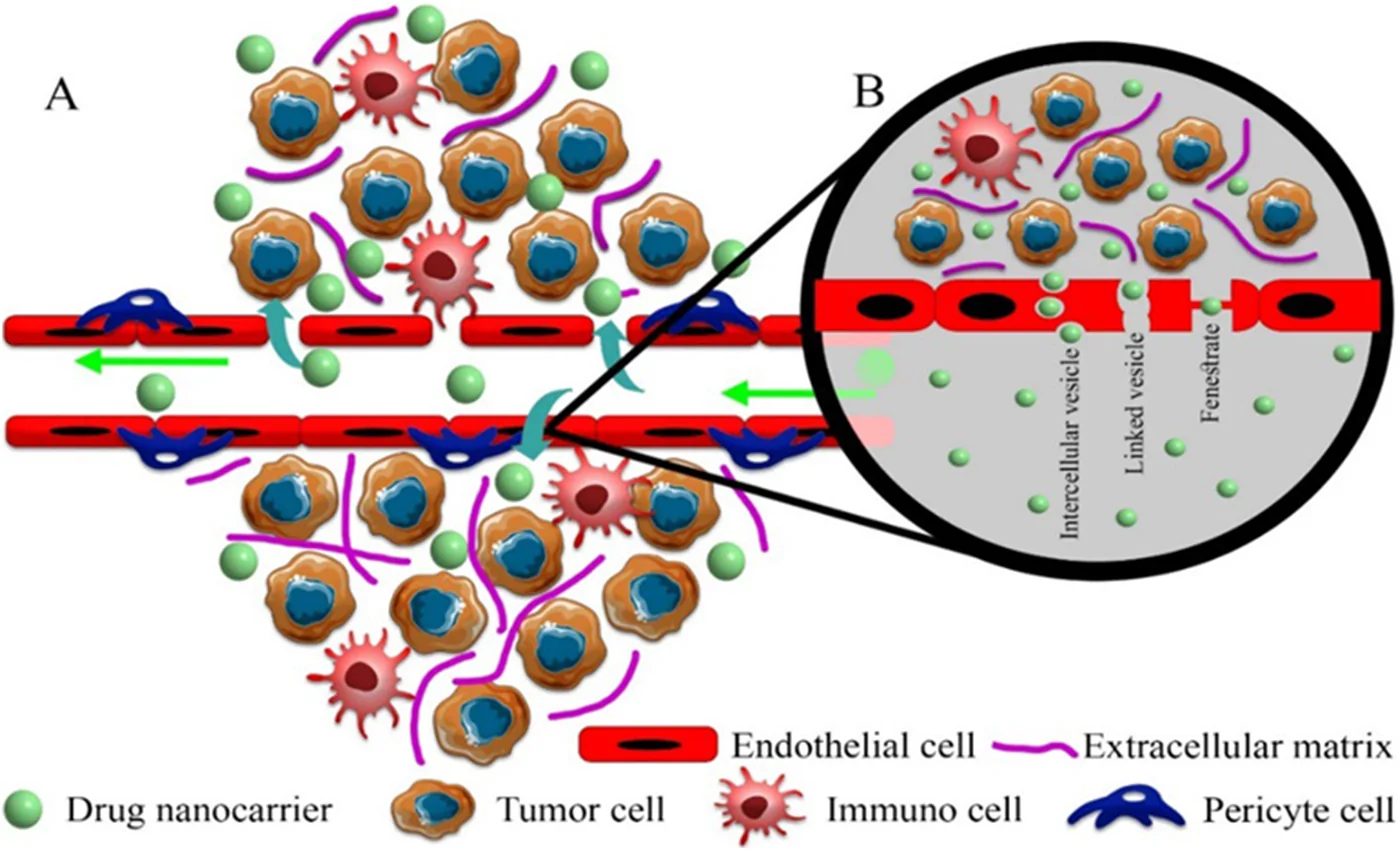
Proposed pathways for drug nanocarriers to enter solid tumors. **(A)** Paracellular process: in this pathway, drug nanocarriers passively enter the extracellular space of solid tumors through intercellular gaps with dimensions up to 2000 nm, which are very important in the EPR effect. **(B)** Transcellular process: drug nanocarriers in mature vessels without common gaps in solid tumors actively enter the extracellular space of the solid tumor through vesicles (endocytosis-exocytosis) and pores. The fenestrate pathway does not have a complete incision, and a diaphragm separates the internal space from the lumen of the vessel. In the linked vesicle path, interconnected vesicles cause the transfer of drug nanocarriers. Reprint with permission from (Majid Sharifi), (An Updated Review on EPR-Based Solid Tumor Targeting Nanocarriers for Cancer Treatment); published by (MDPI), (2022) (Sharifi et al., 2022).

Nanocarriers (liposomes, polymeric nanoparticles, and emerging carbon-based structures) attempt to solve both problems by concentrating drug at the tumor site (via passive EPR effect and active targeting), facilitating controlled release within tumor tissue, and enabling co-delivery of imaging or adjuvant modalities (e.g., photosensitizers) to permit image guided therapy or combined modalities (chemotherapy + photothermal/photodynamic therapy). For an anatomically complex organ such as the gallbladder in biliary tract cancer, where surgical access may be limited by adjacent liver invasion; theranostic nanocarriers that allow intraoperative imaging, tumor margin delineation, and localized therapy hold particular appeal (Kaurav et al., 2023). Among the diverse nanoplatforms investigated, carbon dots (CDs) and carbon quantum dots (CQDs) have attracted considerable attention because they combine excellent biocompatibility, facile surface functionalization, fluorescence imaging capability, and multimodal therapeutic potential. These unique characteristics make them promising candidates for the diagnosis and treatment of BTC and form the focus of the following sections.

## 3 Carbon dots and carbon quantum dots: structural evolution and functional advantages

Carbon dots (CDs) are a class of carbon-based nanomaterials with sizes typically below 10 nm. Their high photostability, biocompatibility, and surface functionality make them promising candidates for cancer therapy, acting as drug carriers, imaging probes, and phototherapeutic agents (Bayda et al., 2021). The fluorescent carbon-based nanoparticles commonly referred to as carbon quantum dots (CQDs) were introduced in the literature by Sun and co-workers in 2006, who described a synthesis that produced highly emissive particles through surface passivation. Since then, nomenclature in the field has expanded in terms such as carbon nanoclusters or carbon dots (CDs) have been used to describe related architectures with varying structures and surface chemistries. Interest in these carbon nanomaterials has grown sharply because of their distinctive optical and surface properties; historically, the generic label “carbon dots” was applied broadly without detailed distinction of morphology or composition (Sun et al., 2006). The evolution from conventional carbon dots (CDs) to carbon quantum dots (CQDs) represents a critical advancement in the structural and functional understanding of carbon-based nanomaterials. Initially, CDs were broadly defined as amorphous or partially graphitized carbon nanoparticles (<10 nm) exhibiting intrinsic fluorescence, often arising from surface defects or molecular fluorophores. However, with progressive refinement in synthesis (e.g., hydrothermal, pyrolytic, or electrochemical routes) and advanced characterization techniques such as HRTEM, XRD, XPS, and Raman spectroscopy, researchers were able to delineate a more structurally ordered subclass which was carbon quantum dots. CQDs possess a distinct graphitic or sp^2^-hybridized crystalline core surrounded by abundant oxygen- or nitrogen-containing surface functional groups, providing well-defined electronic band structures and quantum confinement effects. This transition from structurally heterogeneous CDs to highly crystalline CQDs has enabled precise modulation of photoluminescence behavior, improved photostability, and enhanced biocompatibility. Consequently, CQDs are now recognized as a refined generation of carbon nanodots with superior optical tunability and functionality, underpinning their expanding role in bioimaging, targeted drug delivery, and photodynamic or photothermal cancer therapies (Cai et al., 2021). Figure 4 depicts the evolutional stages from carbon dots to carbon quantum dots (Pang, 2022).

**FIGURE 4:**
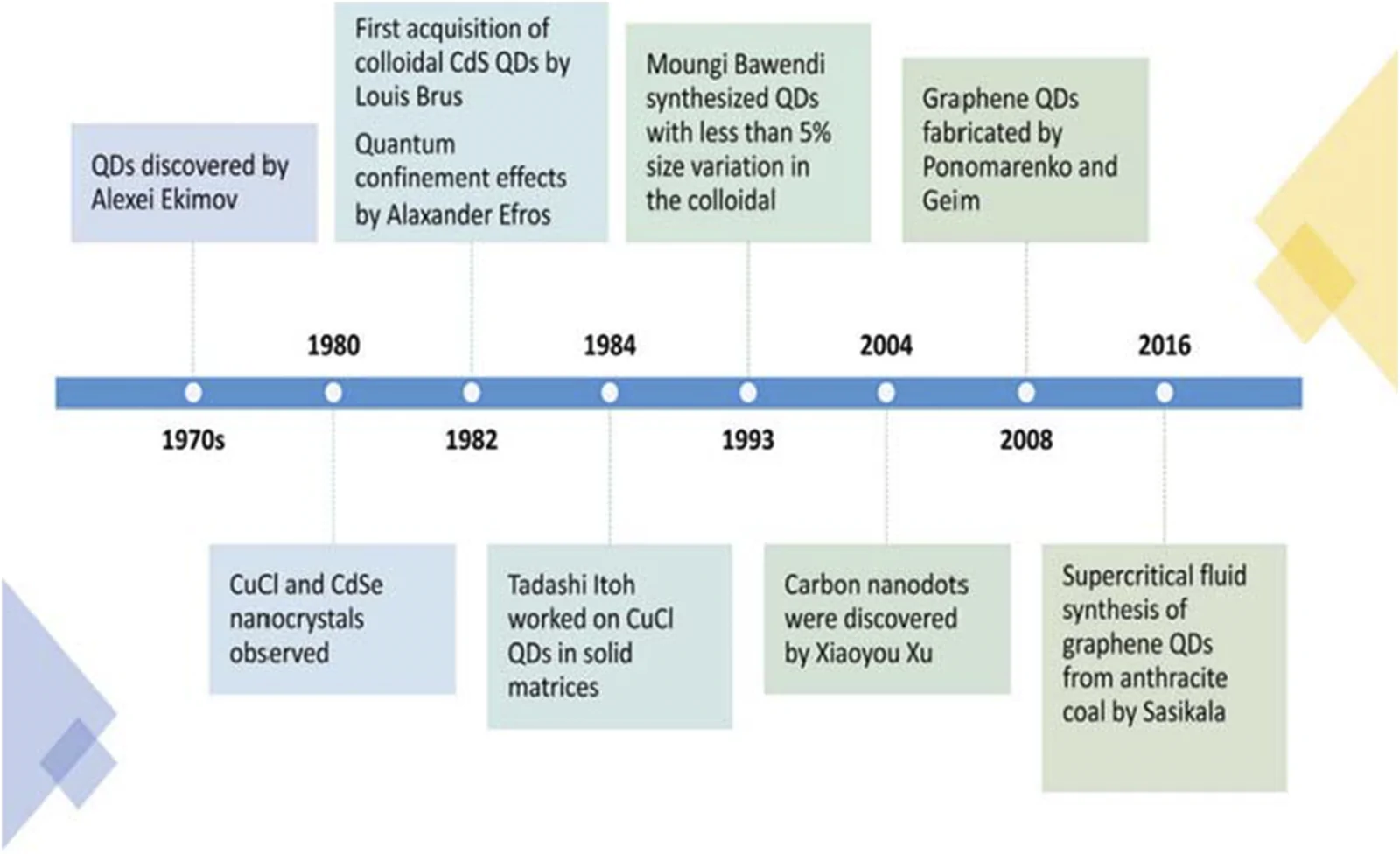
The development history of CQDs. Reprint with permission from (Pang Y) (The synthesis of carbon-based quantum dots: A supercritical fluid approach and perspective); published by (Elsevier) (2022) (Pang, 2022).

### 3.1 Properties and advantages of carbon dot

Carbon dots typically consist of a graphitic, sp^2^-rich core enveloped by an outer layer bearing abundant functional groups (e.g., carboxyl, amino, hydroxyl). This core-shell architecture gives rise to π–π conjugation within the core while the functionalized surface allows facile chemical modification and conjugation. Key attributes that make CDs attractive for biomedical uses include strong and tunable photoluminescence, good aqueous dispersibility, relatively low cytotoxicity, and versatile surface chemistry that supports drug loading, targeting ligand attachment, or further derivatization (Ge et al., 2014). In photodynamic therapy (PDT) (Sun et al., 2020), CDs have been explored in several roles: as intrinsic photosensitizers, as carriers that deliver conventional photosensitizing agents, and in combination regimens that integrate photothermal therapy (PTT) with chemotherapy to achieve synergistic tumor ablation. Collectively, these physicochemical and functional properties underpin the growing interest in carbon dots as a multifunctional platform for cancer theranostics (Zhan et al., 2023). Carbon Dots combine several favorable traits. Some are discussed below.

#### 3.1.1 Photoluminescence of CDs for imaging

CDs exhibit strong fluorescence that can be tuned from visible to near-infrared wavelengths. This allows concurrent imaging-guided therapy (theranostics). Phototherapies (photodynamic and photothermal), chemodynamic therapy, and combinational regimens further expand CDs’ therapeutic repertoire. Certain doped or surface-modified CDs act as photosensitizers to generate reactive oxygen species under light, enabling localized tumor ablation with minimal systemic effects; others convert light into heat for photothermal destruction. Combining these modalities with chemotherapy (drug loading) on the same CD platform has yielded synergistic tumor suppression in animal models, indicating a promising route to potent, localized treatments that could be particularly beneficial for anatomically constrained cancers like GBC (Durão et al., 2018).

#### 3.1.2 Biocompatibility and low toxicity of CDs

Carbon dots (CDs) demonstrate remarkable biocompatibility and low toxicity compared to most inorganic nanoparticles, particularly heavy-metal-based quantum dots. Their carbonaceous composition and tunable surface chemistry make them inherently safer for biological applications. The surface passivation and heteroatom doping (such as nitrogen or bromine) not only enhance optical and photophysical properties but also significantly reduce potential cytotoxicity by minimizing surface defects, aggregation, and unintended reactive oxygen species (ROS) formation. Functionalization with hydrophilic and biocompatible groups improves dispersion and cellular compatibility, allowing CDs to interact safely within biological environments. Furthermore, ultra-small CDs, generally below 5 nm in diameter, can be excreted renally, thereby preventing long-term accumulation in tissues, which is a common concern with larger or metal-based nanoparticles. Collectively, these properties make CDs promising candidates for biomedical uses, including imaging, sensing, and phototherapy, where maintaining biological safety is crucial (Ozyurt et al., 2023).

#### 3.1.3 Drug loading capability of CDs

The drug loading mechanism of carbon dots (CDs) primarily depends on their graphitic core and abundant surface functional groups, which provide multiple binding sites for therapeutic molecules. The conjugated π-electron system of the carbon core enables strong π–π stacking interactions with aromatic drug molecules, such as doxorubicin, allowing them to adsorb efficiently onto the CD surface. Additionally, electrostatic interactions play a crucial role as negatively charged carboxyl or hydroxyl groups on CDs can attract and bind positively charged regions of drugs, forming stable yet reversible complexes. Hydrogen bonding and van der Waals forces further contribute to the stabilization of these interactions, particularly with drugs containing polar functional groups. Together, these non-covalent mechanisms allow CDs to exhibit high drug-loading capacities and controlled, often pH-responsive, drug release profiles, making them highly effective and tunable nanocarriers for biomedical applications (Sun et al., 2017). Red-bean-derived carbon dots exhibited significant antiproliferative and antimigratory effects against various cancer cell lines, including the intrahepatic cholangiocarcinoma (RBE) line. Their activity was dose- and time-dependent, showing maximal inhibition at 5 mg/mL. In biliary tract cancer cells, the carbon dots effectively reduced cell growth and motility, highlighting their therapeutic potential. Moreover, they enhanced the cytotoxic efficacy of conventional chemotherapeutics, suggesting synergistic action in biliary tract cancer treatment (Xia et al., 2019). Figure 5 shows the schematic mechanism of the transportation of CDs within the cells.

**FIGURE 5:**
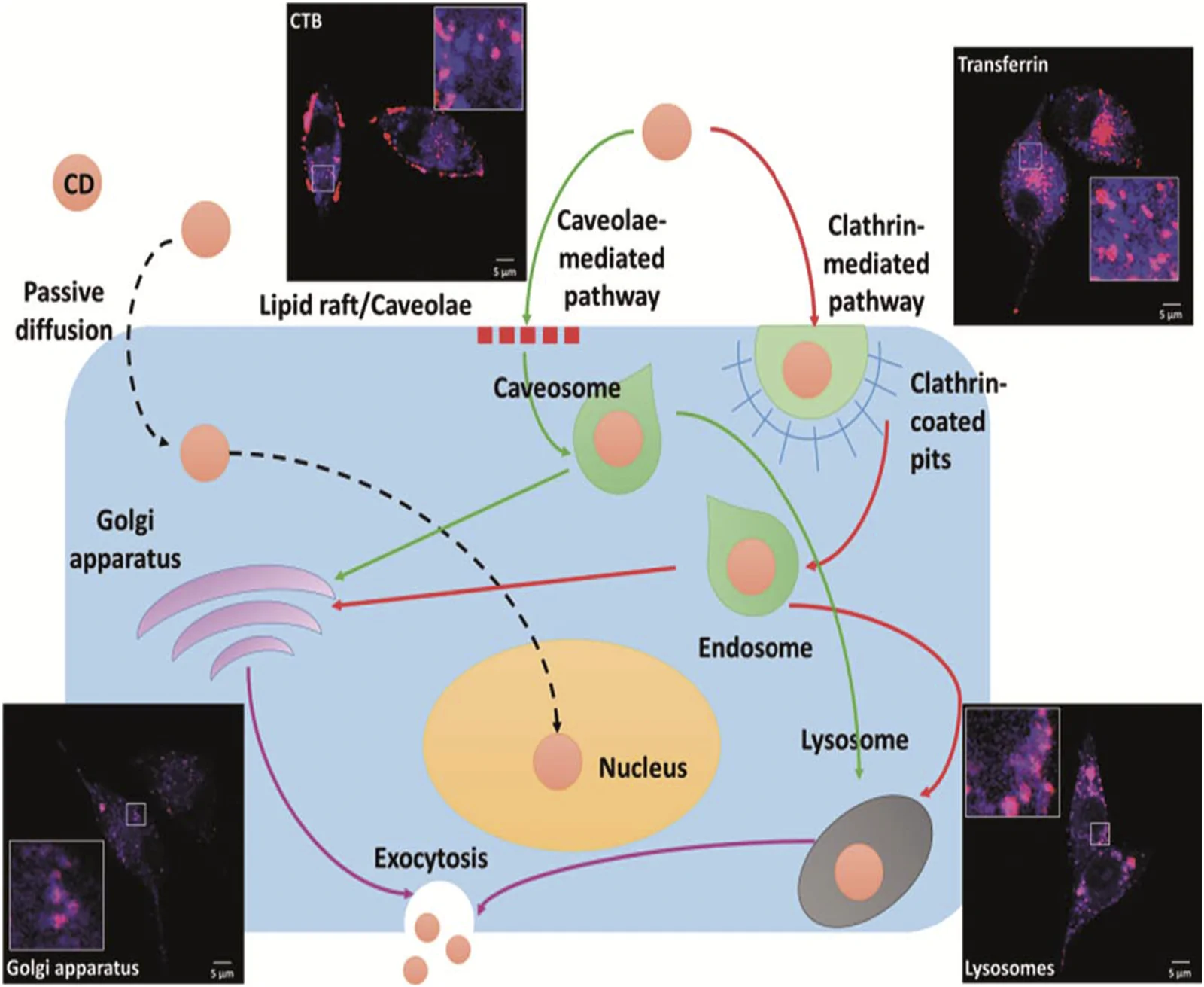
Schematic diagram illustrating the key steps involved in CDs uptake, intracellular trafficking and exocytosis (Zhou et al., 2014). Reprint with permission from (Nan Zhou) (Elucidating the endocytosis, intracellular trafficking, and exocytosis of carbon dots in neural cells); published by (RSC Advances), (2014) (Zhou et al., 2014).

### 3.2 Transition of CD into CQDs and its properties and advantages in biliary tract cancer therapy

Carbon quantum dots (CQDs) represent a structurally advanced subclass of carbon dots distinguished by a highly crystalline carbon core, pronounced quantum confinement effects, and superior optical performance. Although CQDs share the fundamental characteristics of conventional carbon dots, including ultrasmall dimensions, abundant surface functional groups, and excellent aqueous dispersibility, they generally exhibit higher quantum yields, improved photostability, narrower emission spectra, and enhanced fluorescence intensity. These characteristics make CQDs particularly attractive for precision oncology applications requiring simultaneous imaging, targeted drug delivery, and phototherapy. CQDs can be synthesized through relatively simple and cost-effective processes, frequently using bio-based precursors, which makes them attractive for sustainable nanomaterial production. In addition, their excitation and emission wavelengths can be readily tuned, allowing their optical behavior to be tailored for specific applications. CQDs also demonstrate excellent thermal and photostability, ensuring consistent performance under various conditions. Their surfaces can be easily modified through chemical functionalization, enabling the attachment of targeting ligands, drugs, or other functional molecules. Importantly, CQDs generally show minimal cytotoxicity and good biocompatibility, making them promising candidates for biomedical applications such as bioimaging, drug delivery, and theranostics (Chauhan et al., 2022). Different properties of CQDs are discussed below.

#### 3.2.1 Crystallinity and structure

The internal structure or crystallinity of carbon dots plays a crucial role in how they interact with light and biological tissues. Crystalline carbon quantum dots (CQDs) have an orderly graphitic core that allows electrons to move freely, improving their optical and thermal properties. This ordered arrangement enhances their ability to absorb light and convert it into heat or reactive oxygen species (ROS), both essential for light-based cancer treatments (Tavan et al., 2025). In contrast, amorphous carbon dots (CDs) have a more disordered structure, leading to inconsistent energy transfer and lower therapeutic efficiency. In the case of biliary tract cancers such as gallbladder or bile duct cancer, CQDs are particularly advantageous because their predictable heat generation helps destroy cancer cells precisely while minimizing damage to nearby liver tissue. The crystalline structure also contributes to better chemical stability and reproducibility, which are important for clinical translation (Bhattacharya et al., 2023).

#### 3.2.2 Photoluminescence, quantum yield, and imaging potential

Photoluminescence (PL) refers to the ability of carbon dots to emit light after excitation, which is critical for bioimaging and diagnostic applications. A high quantum yield (brightness) and longer emission lifetime make the nanoparticles more visible during imaging or surgery. For biliary tract cancers, imaging is challenging because tumors are deep inside the body, where visible light cannot easily penetrate. CQDs can be engineered to emit near-infrared (NIR) light, which penetrates deeper into tissues than visible wavelengths, enabling clearer imaging of gallbladder or bile duct lesions during endoscopic or surgical procedures (Dimitriev et al., 2024). Amorphous CDs typically emit blue or green light with shallow tissue penetration, limiting their medical usefulness. Therefore, CQDs with their crystalline cores and tunable surface doping offer superior imaging performance, better brightness, and more stable fluorescence under biological conditions, making them more suitable for image-guided BTC diagnosis and treatment (Li et al., 2023). Figure 6 demonstrate the vast applications of CQDs in different fields.

**FIGURE 6:**
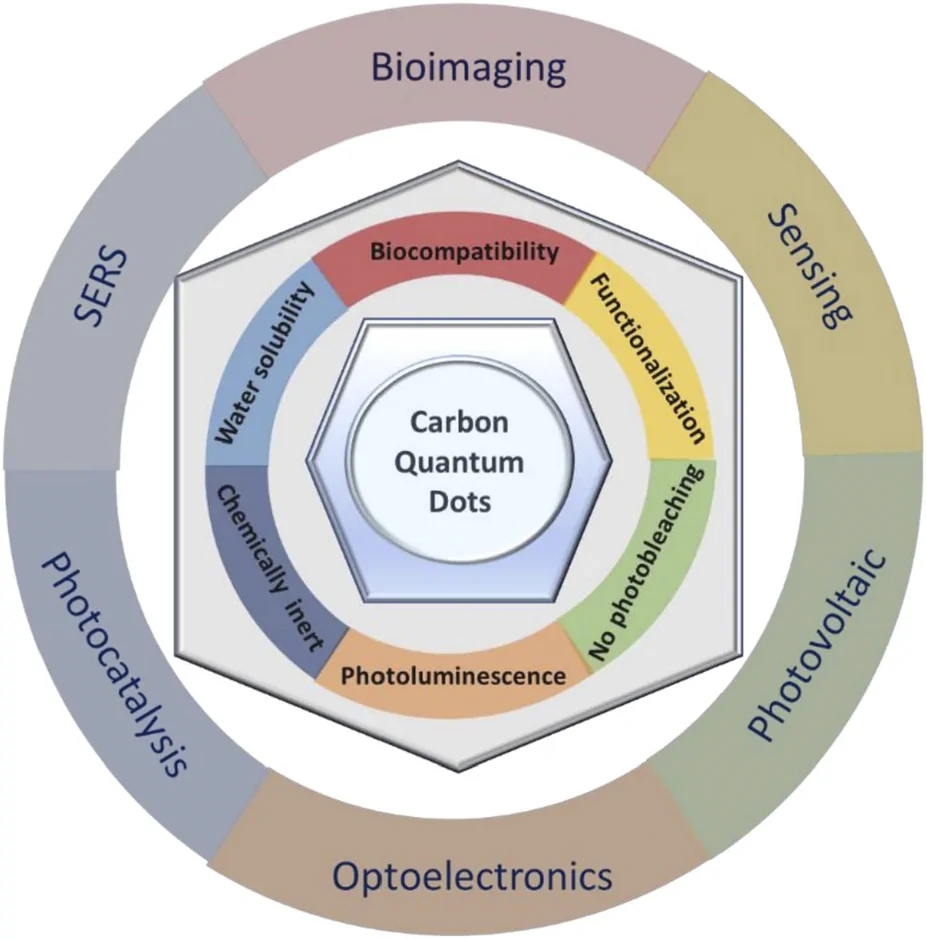
Diverse fields of applications of CQDs (Sousa et al., 2021). Reprint with permission from (Helena B. A. Sousa) (You Don’t Learn That in School: An Updated Practical Guide to Carbon Quantum Dots); published by (MDPI) (2021) (Sousa et al., 2021).

#### 3.2.3 Charge transfer, photovoltaic effect, and photodynamic therapy

When light strikes a carbon quantum dot, it excites electrons and creates electron hole pairs. The ability of these electrons to separate and move is known as charge transfer or the photovoltaic effect. This property determines how efficiently a dot can produce reactive oxygen species (ROS), which are toxic molecules that kill cancer cells in photodynamic therapy (PDT) (Sun et al., 2006). In biliary tract cancers, light can be delivered through fiber optics during minimally invasive procedures. Materials that can generate ROS efficiently at low light doses are safer and more effective in such treatments. CQDs excel in this regard because their crystalline cores and controlled doping improve charge separation and reduce electron recombination. This means CQDs can produce more ROS than amorphous CDs under the same light exposure, resulting in stronger cancer cell killing with less damage to surrounding tissues (Aebisher et al., 2024).

#### 3.2.4 Photothermal conversion and controlled heating

Photothermal therapy (PTT) relies on nanoparticles that can efficiently convert light energy into heat, selectively destroying tumor cells. The ability of a material to generate heat upon illumination is called photothermal conversion efficiency. CQDs, because of their crystalline nature and abundant non-radiative energy pathways, can convert light into heat more effectively and more predictably than amorphous CDs (Cui et al., 2023). This property is highly valuable in treating biliary tract cancers, where tumors are close to delicate structures like bile ducts and liver tissue, and controlled heating is critical to avoid collateral damage. CQDs can achieve effective tumor ablation with lower laser power, while CDs may produce uneven heating or weaker effects. Consequently, CQDs offer safer, more efficient, and better-controlled thermal performance in PTT or combined PDT–PTT approaches for BTC (Board, 2025).

#### 3.2.5 Surface chemistry, size, and biliary clearance

The biological safety and effectiveness of carbon dots depend strongly on their size, surface charge, and surface functional groups These characteristics influence how the nanoparticles move through the body, accumulate in the liver or bile ducts, and are ultimately excreted (Truskewycz et al., 2022). For BTC therapy, it is vital to control these parameters because excessive accumulation in the liver or bile ducts may cause toxicity or obstruction. CQDs can be synthesized with precise size control and well-defined surface coatings, allowing researchers to design particles that either stay near the tumor site for localized treatment or clear quickly through the kidneys to avoid long-term accumulation. Amorphous CDs often show wide size variation and unstable surface chemistry, making their behavior in the body unpredictable. Therefore, CQDs provide more reliable control over biodistribution and biliary excretion, improving safety and therapeutic targeting for gallbladder and bile duct cancers (Lu, 2016).

### 3.3 Carbon quantum dots have emerged as a preferred alternative to CDs and two-dimensional carbon nanomaterials (GO and rGO)

Carbon dots (CDs) and carbon quantum dots (CQDs) have increasingly attracted attention as alternatives to two-dimensional carbon nanomaterials such as graphene oxide (GO) and reduced graphene oxide (rGO) for cancer theranostics. Although GO and rGO possess exceptionally high surface area and excellent drug-loading capacity through π–π interactions, their relatively large lateral dimensions, poor biodegradability, prolonged tissue retention, and potential induction of oxidative stress have raised concerns regarding long-term biosafety. In contrast, CDs are ultrasmall (<10 nm), highly water-dispersible nanoparticles that exhibit excellent photoluminescence, facile renal clearance, lower immunogenicity, and reduced accumulation in the reticuloendothelial system (Parvin et al., 2024). Another major advantage of CDs lies in their intrinsic fluorescence, which enables simultaneous imaging and drug delivery without additional fluorescent labels (Molaei, 2019). While GO generally requires external fluorophores or imaging probes, CDs inherently function as theranostic agents by integrating diagnosis and therapy into a single nanoplatform. Furthermore, the abundant surface functional groups of CDs facilitate straightforward conjugation with targeting ligands, chemotherapeutic drugs, nucleic acids, and photosensitizers while preserving colloidal stability (Parvin et al., 2024). For biliary tract cancers, where efficient penetration through dense desmoplastic stroma and rapid systemic clearance are desirable, the ultrasmall size of CDs offers improved tumor penetration compared with micron-scale GO sheets. In addition, CDs exhibit superior photothermal conversion and reactive oxygen species generation following heteroatom doping, making them attractive candidates for combined photothermal-photodynamic therapy. Nevertheless, GO and rGO continue to possess advantages for high-capacity drug loading and certain photothermal applications, indicating that the optimal nanocarrier depends on the intended therapeutic objective. Overall, the favorable balance between biosafety, multifunctionality, fluorescence imaging, and controllable surface chemistry has positioned CDs as one of the most promising carbon-nanoplatforms for next-generation precision oncology (Javan et al., 2026). Table 1 shows Comparison of graphene oxide (GO), reduced graphene oxide (rGO), carbon dots (CDs), and carbon quantum dots (CQDs) for cancer theranostic applications, with particular relevance to biliary tract cancer (BTC).

**TABLE 1:** Comparison of graphene oxide (GO), reduced graphene oxide (rGO), carbon dots (CDs), and carbon quantum dots (CQDs) for cancer theranostic applications, with particular relevance to biliary tract cancer (BTC).

Sr No.	Parameter	Graphene oxide (GO)	Reduced graphene oxide (rGO)	Carbon dots (CDs)	Carbon quantum dots (CQDs)	Ref.
1	Typical size	Lateral dimensions typically 100 nm–several μm; thickness ∼1 nm	Similar lateral dimensions to GO; thickness ∼1–5 nm	Typically <10 nm	Typically 2–10 nm	Hong et al. (2022) (Molaei (2019)
2	Structure	Two-dimensional oxidized graphene sheet rich in oxygen-containing functional groups	Partially reduced graphene sheet with restored sp2 domains and fewer oxygen groups	Quasi-spherical amorphous or nanocrystalline carbon nanoparticles	Quasi-spherical crystalline carbon nanoparticles with graphitic core	Hong et al. (2022)
3	Drug-loading capacity	Very high via π–π stacking, hydrophobic interactions, and hydrogen bonding	High; strong π–π interactions due to restored graphitic domains	Moderate to high through abundant surface functional groups and covalent conjugation	Moderate to high; suitable for covalent conjugation and electrostatic interactions	Molaei (2019)
4	Intrinsic fluorescence	Weak or absent; usually requires fluorescent labeling	Very weak fluorescence	Strong excitation-dependent fluorescence	Strong and stable fluorescence with high quantum yield	Molaei (2019)
5	Surface functionalization	Excellent due to abundant hydroxyl, epoxy, and carboxyl groups	Good, although fewer oxygen-containing groups than GO	Excellent; rich in amino, hydroxyl, and carboxyl groups enabling facile bioconjugation	Excellent; easily modified with polymers, peptides, antibodies, nucleic acids, and drugs	Molaei (2019)
6	Water dispersibility	High	Moderate; often requires additional stabilizers	Excellent	Excellent	Molaei (2019)
7	Biocompatibility	Moderate; dependent on 8oxidation degree, size, and dose	Moderate to good after appropriate surface modification	Excellent in most reported in vitro and in vivo studies	Excellent with generally low cytotoxicity	Molaei (2019)
8	Potential toxicity	May induce oxidative stress, inflammation, membrane damage, and prolonged tissue retention at high doses	Lower than GO after functionalization but still associated with dose-dependent oxidative stress	Generally low toxicity and low immunogenicity	Low toxicity with favorable biosafety profile	Parvin et al. (2024)
9	Biodistribution and clearance	Slow clearance; accumulation in liver, spleen, and lungs is common	Slow clearance with possible long-term organ retention	Efficient renal clearance owing to ultrasmall size	Efficient renal clearance with reduced long-term accumulation	Parvin et al. (2024)
10	Photothermal capability	Good	Excellent due to restored electronic conductivity	Moderate to excellent after heteroatom doping or hybridization	Moderate to excellent depending on composition and doping	Parvin et al. (2024)
11	Photodynamic (ROS) generation	Limited unless combined with photosensitizers	Moderate	High after heteroatom doping and surface engineering	High with appropriate surface engineering	D’Acapito et al. (2023) Parvin et al. (2024)
12	Cellular uptake and tumor penetration	Limited by relatively large sheet dimensions	Moderate	Excellent because of ultrasmall size	Excellent because of ultrasmall size	Molaei (2019)
13	Imaging capability	Requires external imaging probes	Limited intrinsic imaging capability	Simultaneous fluorescence imaging and therapy (theranostic platform)	High-performance fluorescence imaging with potential for multimodal imaging	Molaei (2019)
14	Clinical translation potential	Limited by biosafety and biodegradation concerns	Limited; requires further toxicity evaluation	High owing to favorable safety, multifunctionality, and facile synthesis	High owing to excellent optical properties and biocompatibility	Javan et al. (2026)
15	Suitability for biliary tract cancer (BTC)	Effective for high-capacity drug delivery but limited by large size and prolonged retention in dense stromal tumors	Useful for photothermal therapy but long-term safety remains under investigation	Highly suitable because of superior tumor penetration, fluorescence-guided imaging, renal clearance, and easy functionalization	Highly suitable for image-guided targeted therapy, biosensing, and multimodal theranostics	Javan et al. (2026)

## 4 Targeting mechanisms of carbon dots for BTC therapy

CD nanocarriers can exploit both passive and active targeting in BTC:

### 4.1 Passive targeting (EPR effect)

Tumors often have leaky vasculature and poor lymphatic drainage. Nanoscale CDs naturally accumulate in tumors via the enhanced permeability and retention (EPR) effect. This effect is present in BTC, especially intrahepatic CCA. Passive uptake increases local drug concentration relative to free drug (Subhan et al., 2021). Different types of nano-carriers for active and passive tumour targeting are shown in Figure 7.

**FIGURE 7:**
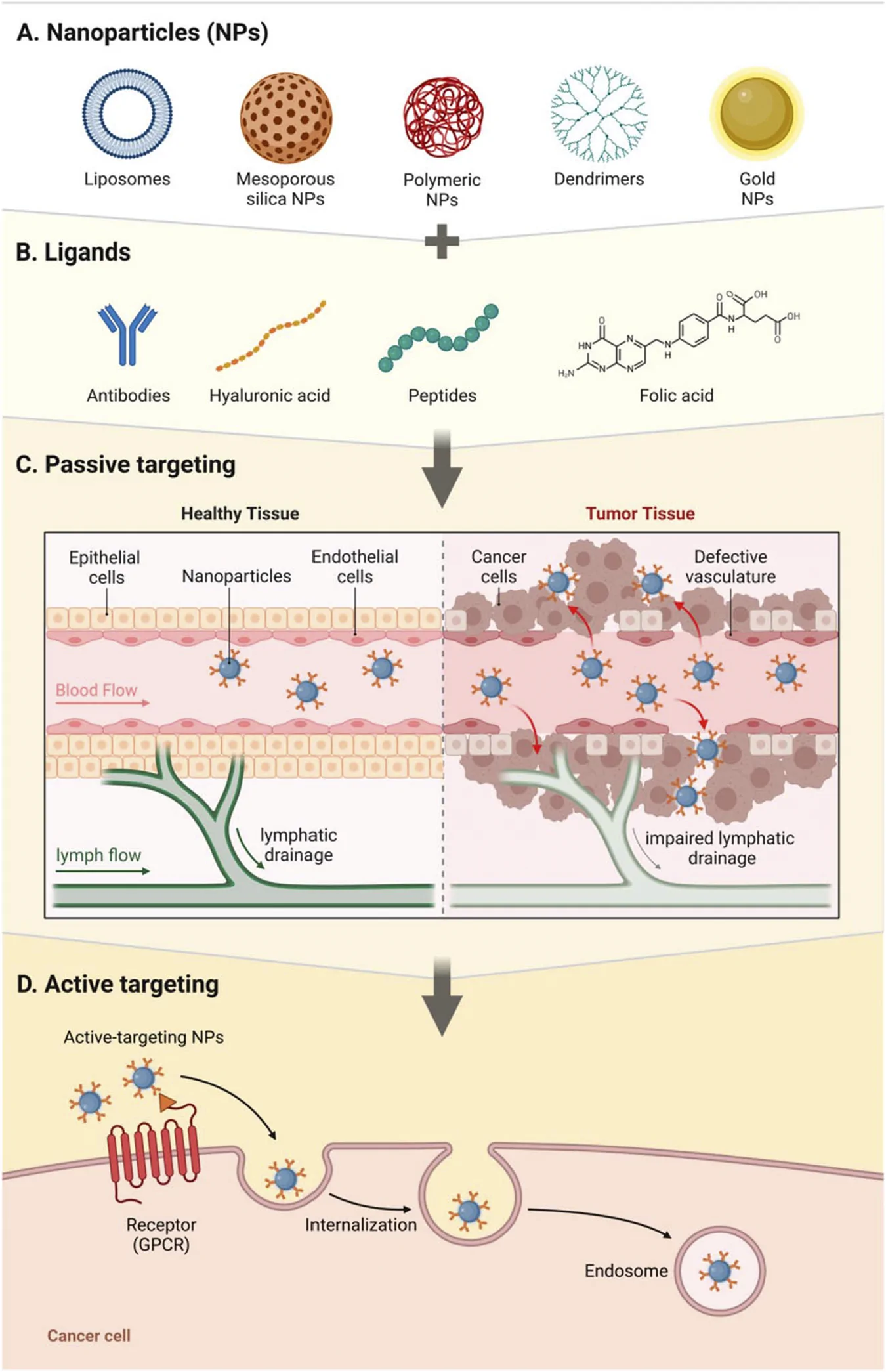
Schematic illustration of the different types of nanoparticles used for passive and active tumor targeting through the EPR effect (Vagena et al., 2025). Reprint with permission from (Ioanna-Aglaia Vagena), (Enhancement of EPR Effect for Passive Tumor Targeting: Current Status and Future Perspectives); published by (MDPI), (2025) (Vagena et al., 2025).

### 4.2 Active targeting (ligand functionalization)

CDs can be decorated with targeting ligands to bind overexpressed receptors on BTC cells. For example,: RGD peptides target integrin α vβ 3 (overexpressed in some liver metastases) (Li et al., 2019). Folate can target folate receptors on cancer cells. Hyaluronic acid (HA) targets CD44; epithelial cell adhesion molecule (EpCAM) is highly expressed in cholangiocarcinoma (Chehelgerdi et al., 2023). In each case, ligand CD binding enhances uptake by tumor cells and reduces off-target effects. For instance, RGD-modified CDs achieved strong uptake in α_vβ_3-positive cells (Li et al., 2019).

### 4.3 Active targeting stimuli triggered release

CDs can also incorporate cleavable linkers or photosensitizer moieties for on-site drug release. Li et al. developed a multifunctional nanocarrier based on carbon quantum dots (CQDs) that co-deliver the chemotherapeutic drug Doxorubicin (DOX) and the photosensitizer 5-Aminolevulinic acid (5-ALA). The system was characterized (size, fluorescence, drug loading) and evaluated *in vitro* against cancer cells. The authors found high DOX-encapsulation efficiency (83%) and demonstrated pH‐dependent drug release, with 91% of 5-ALA released at acidic pH (2.8) over 24 h, and 41.25% of DOX in the same timeframe. Under laser irradiation, the nanocarrier produced enhanced reactive oxygen species (ROS) generation, leading to greater cancer-cell damage compared to chemotherapy or photodynamic therapy alone. The combined chemo‐PDT approach showed synergistic anticancer efficacy in the tested cancer cell line. The research work conclude that their CQD-based carrier offers controllable drug release, imaging/fluorescence capability, and a dual therapeutic modality, making it a promising candidate for enhanced cancer therapy (Li et al., 2020). The overall schematic representation for the preparation of 5-ALA-CQD-Glu-β-CD nanocarrier presented in Figure 8.

**FIGURE 8:**
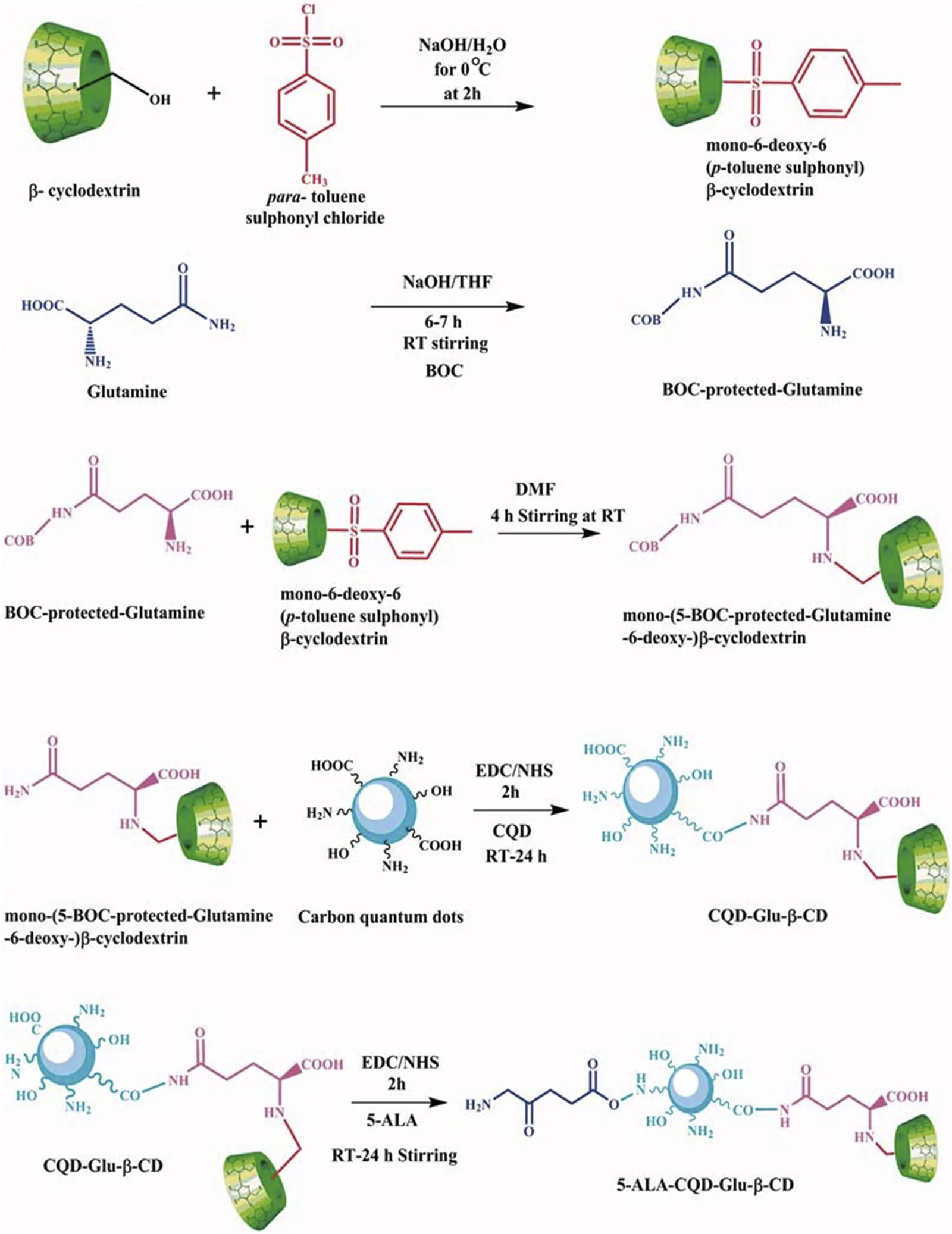
Overall representation for the preparation of 5-ALA-CQD-Glu-β-CD nanocarrier (Li et al., 2020). Reprint with permission from (Xin Li), (Combined photodynamic-chemotherapy investigation of cancer cells using carbon quantum dot-based drug carrier system); published by (Tylor & Francis), (2020) (Li et al., 2020).

### 4.4 CDs versus CQDs for targeting mechanism

Targeted CDs concentrate drugs in tumors, meaning higher antitumor efficacy with lower systemic toxicity. It is noted that DOX-loaded amino acid mimetic CDs produced superior tumor inhibition *and* minimal side effects compared to conventional liposomal DOX. This target or clear paradigm where particles either hit tumor or are rapidly cleared minimizes normal tissue exposure. CDs’ innate fluorescence also allows clinicians to track distribution and confirm tumor targeting of biliary tract noninvasively (Xie et al., 2025). Carbon quantum dots (CQDs) demonstrate significant advantages over other nanomaterials such as metal quantum dots, carbon nanotubes, and inorganic nanoparticles, making them highly attractive for biomedical applications. Owing to their carbon-based composition and absence of heavy metals, CQDs exhibit low cytotoxicity and excellent biocompatibility. Their abundant surface functional groups (–COOH, –OH, –NH_2_) confer superior water solubility and enable facile chemical modification for conjugation with therapeutic agents or targeting ligands. Additionally, CQDs possess remarkable photostability and tunable fluorescence, allowing their simultaneous use in real-time bioimaging and theranostic applications. Their stimuli-responsive nature enables controlled drug release under tumor-specific conditions such as acidic pH, redox gradients, or light irradiation. Furthermore, CQDs can be synthesized cost-effectively and sustainably from natural precursors like citric acid, glucose, or red beans using simple hydrothermal or microwave-assisted methods. Collectively, these attributes position CQDs as a safe, stable, and multifunctional nanoplatform for targeted, image-guided, and combination cancer therapies (Mondal et al., 2025).

In a recent research work, highly luminescent carbon dots (CDs) with an exceptional fluorescence quantum yield of approximately 97% were synthesized and subsequently functionalized with folic acid (FA) to obtain a targeted imaging and drug-delivery nanoplatform. The CDs exhibited excellent biocompatibility and strong, stable fluorescence suitable for cellular and *in vivo* imaging applications. Doxorubicin (DOX) was successfully loaded onto FA-CDs, forming FA-CDs–DOX, which demonstrated controlled and selective drug-release behavior. *In vitro* assays confirmed efficient uptake of the FA-modified CDs by folate receptor, overexpressing liver cancer cells, enabling clear intracellular fluorescence visualization under confocal microscopy. *In vivo* experiments further showed that FA-CDs–DOX produced high intensity fluorescence signals capable of penetrating skin and tumor tissue, supporting their utility for deep-tissue tumor imaging. Importantly, FA-CDs–DOX exhibited significantly enhanced antitumor efficacy compared with free DOX, as demonstrated by greater tumor-growth inhibition in animal models (Wang S, 2020). Figure 9 explains the application of carbon dots on HepG2 cell lines.

**FIGURE 9:**
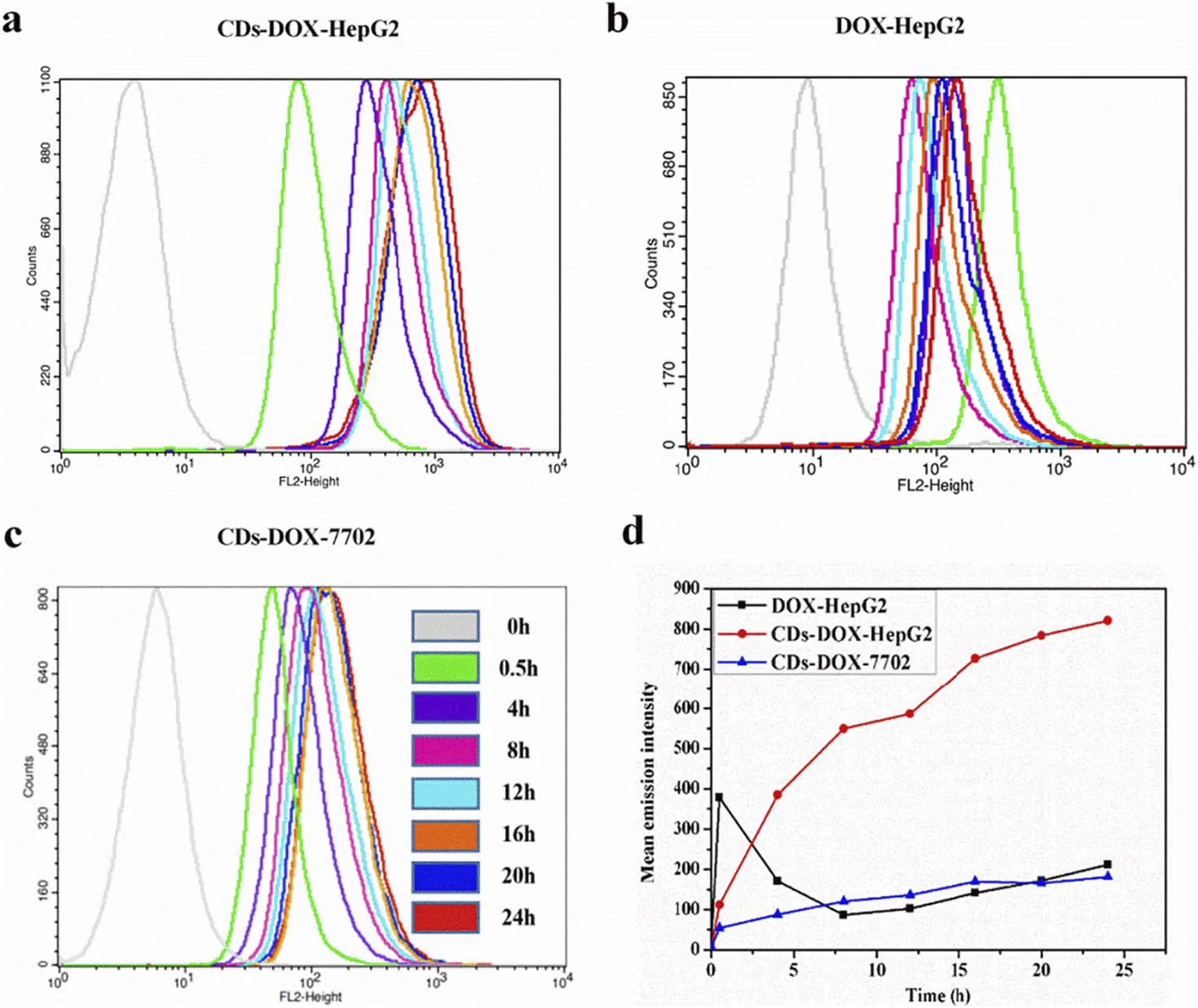
Intracellular release of CDs-DOX: intracellular release of DOX from CDs-DOX **(a)** and free DOX **(b)** in the HepG2 cells; **(c)** intracellular release of DOX from CDs-DOX in 7,702 cells; **(d)** intensity comparison of the release amount of DOX (Wang S, 2020). Reprint with permission from (Shicai Wang), (Enhanced-fluorescent imaging and targeted therapy of liver cancer using highly luminescent carbon dots-conjugated foliate); published by (Elsevier), (2020) (Wang S, 2020).

### 4.5 Photothermal therapy using carbon dots: mechanisms, advantages over graphene-based nanomaterials, and potential applications in biliary tract cancer

Photothermal therapy (PTT) has emerged as a promising minimally invasive strategy for treating solid tumors by converting near-infrared (NIR) light into localized heat that induces irreversible cancer cell death. Unlike conventional chemotherapy, which is often associated with systemic toxicity, PTT enables spatially controlled thermal ablation with minimal damage to surrounding healthy tissues. Its therapeutic efficacy depends on the photothermal conversion efficiency of the nanomaterial, efficient tumor accumulation, adequate NIR tissue penetration, and selective heat generation within tumor tissues (ChenLiu et al., 2025). Carbon-based nanomaterials have been extensively explored as photothermal agents because of their broad optical absorption, chemical stability, and biocompatibility. Among them, graphene oxide (GO), reduced graphene oxide (rGO), carbon dots (CDs), and carbon quantum dots (CQDs) efficiently convert absorbed NIR light into heat through non-radiative relaxation. GO and rGO exhibit high photothermal conversion efficiency owing to their extended π-conjugated structures and large surface area, which also facilitates drug loading for combined chemo-photothermal therapy (Wang J, 2020). However, their relatively large dimensions, limited biodegradability, physiological aggregation, and prolonged hepatic and splenic retention raise concerns regarding long-term biosafety, particularly for hepatobiliary applications (Karagianni et al., 2025).

In contrast, CDs and CQDs possess ultrasmall particle sizes (<10 nm), excellent aqueous dispersibility, abundant surface functional groups, and favorable pharmacokinetics, enabling efficient tumor penetration and facile functionalization with targeting ligands. Heteroatom doping (e.g., N, S, P, B, or transition metals) further enhances NIR absorption and photothermal conversion while preserving their intrinsic fluorescence, allowing simultaneous imaging and therapy within a single theranostic platform (Tavan et al., 2025). Moreover, many CDs generate reactive oxygen species (ROS) under light irradiation, enabling synergistic photothermal and photodynamic effects that promote mitochondrial dysfunction, DNA damage, membrane disruption, and apoptosis while reducing the laser power and drug doses required for effective tumor ablation (Balou et al., 2022). These multifunctional properties are particularly attractive for biliary tract cancers (BTCs), including gallbladder cancer and cholangiocarcinoma, which are characterized by dense desmoplastic stroma, hypoxia, poor vascularization, and resistance to systemic therapies (Valle et al., 2021). The proposed mechanism underlying CD-mediated photothermal therapy in biliary tract cancer is illustrated in Figure 10.

**FIGURE 10:**
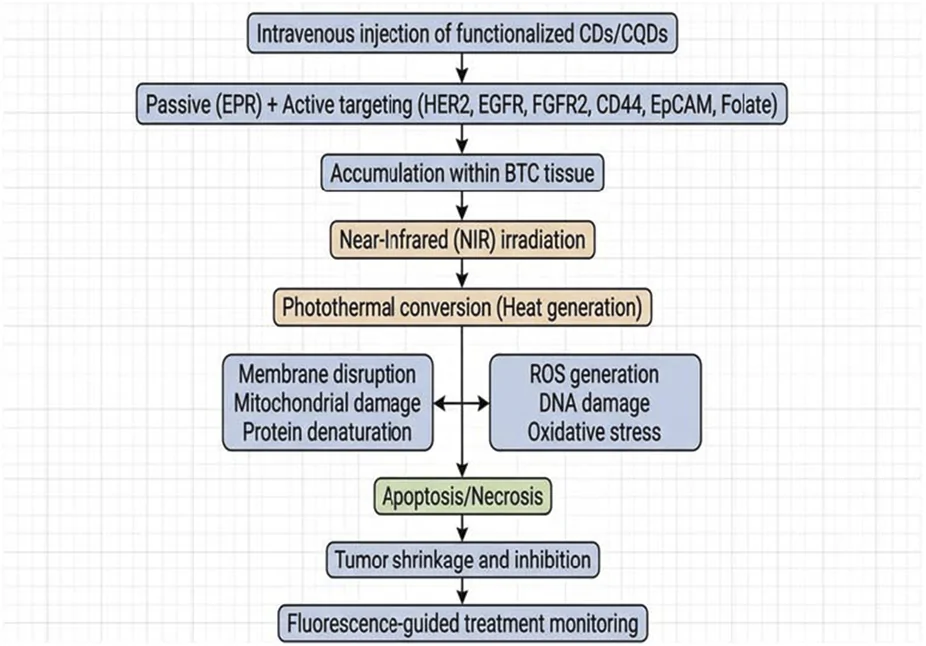
Mechanistic overview of carbon dot-mediated photothermal therapy in biliary tract cancer.

Surface modification of CDs with ligands targeting HER2, EGFR, FGFR2, EpCAM, CD44, or folate receptors can improve tumor-specific accumulation, while NIR irradiation delivered intraoperatively or through endoscopic retrograde cholangiopancreatography (ERCP) offers the potential for localized treatment of otherwise inaccessible biliary tumors (Chakrabarti et al., 2020). Furthermore, PTT-induced hyperthermia can enhance vascular permeability and extracellular matrix remodeling, improving the intratumoral delivery of chemotherapeutic, immunotherapeutic, or photodynamic agents and supporting multimodal treatment strategies (Xu et al., 2025). Despite encouraging preclinical outcomes, several challenges remain before clinical translation. Limited penetration of NIR-I light (700–900 nm), the need for standardized evaluation of photothermal efficiency and biodistribution, and insufficient long-term safety data remain significant barriers (Ying et al., 2025). Future research should focus on NIR-II-responsive CDs, improved tumor-targeting strategies, and validation in orthotopic biliary tract cancer models. Overall, compared with GO and rGO, CDs and CQDs provide a more favorable combination of photothermal efficiency, fluorescence imaging capability, biosafety, and multifunctionality, making them promising nanotheranostic platforms for precision treatment of biliary tract cancers (Karagianni et al., 2025) (Valle et al., 2021). A comparative overview of GO, rGO, CDs, and CQDs in Table 2 highlights the advantages and limitations of each nanomaterial for photothermal therapy and theranostic applications in biliary tract cancers.

**TABLE 2:** Comparative characteristics of carbon-based nanomaterials used for photothermal therapy.

Parameter	GO	rGO	CDs	CQDs	Ref.
NIR absorption	Excellent	Excellent	Good	Excellent	Ying et al. (2025)
Photothermal conversion	High	High	Moderate–High	High	ChenLiu et al. (2025)
ROS generation	Moderate	Moderate	High	High	Valle et al. (2021)
Fluorescence imaging	Poor	Poor	Excellent	Excellent	Xu et al. (2025)
Drug loading	Excellent	Excellent	Good	Good	Karagianni et al. (2025)
Surface modification	Good	Good	Excellent	Excellent	Balou et al. (2022)
Tumor penetration	Moderate	Moderate	Excellent	Excellent	Valle et al. (2021)
Renal clearance	Poor	Poor	High	High	Tavan et al. (2025)
Long-term biosafety	Moderate	Moderate	High	High	Tavan et al. (2025)
BTC translational potential	Moderate	Moderate	High	Very High	Xu et al. (2025)

## 5 Molecular and developmental parallels between gallbladder and other biliary cancers biliary tract cancers: gallbladder in context

To this date, no study has specifically examined carbon dot therapy in gallbladder carcinoma. Therefore, the inferences must be drawn from related hepatobiliary cancers. The gallbladder is anatomically and developmentally linked to the liver’s biliary system (Moeini et al., 2021). Bile produced by the liver travels through bile ducts to the gallbladder for storage. Gallbladder mucosal cells and bile-duct cholangiocytes share a common embryonic origin. In fact, genomic analyses show that gallbladder cancer (GBC) often shares mutations (e.g., TP53, HER2 amplification) with cholangiocarcinoma (CCA) and hepatocellular carcinoma (Housset et al., 2016). Clinically, GBC and CCA also present similar symptoms (jaundice, pain) and risk factors (chronic inflammation, gallstones).

This close relationship suggests that success in CD-based therapies for liver and bile-duct tumors could translate to gallbladder cancer. For instance, cholangiocarcinoma (bile duct cancer) has a very poor prognosis (5 years with survival rate of only 9%) (Shen et al., 2022). A 2023 review emphasized that nanoplatforms (including carbon dots) may be crucial for improving cholangiocarcinoma treatment by enhancing drug efficacy and reducing side effects. By extension, similar nano-strategies might benefit GBC patients (Liu et al., 2023).

### 5.1 Molecular targets

While specific targeted drugs for gallbladder cancer (GBC) remain limited, the Oyasiji et al. (2015) discusses several actionable molecular pathways shared across biliary tract cancers. These include FGFR2 fusions predominantly found in intrahepatic cholangiocarcinoma, and HER2 amplifications or overexpression observed in extrahepatic cholangiocarcinoma and GBC. Other implicated pathways involve EGFR, RAS–RAF–MEK–ERK, and PI3K–AKT–mTOR, all representing potential therapeutic targets under clinical exploration. Building on these molecular insights, nanocarriers such as carbon dots (CDs), Carbon Quantum Dots could be engineered to deliver targeted agents or conventional chemotherapeutics precisely to tumor sites. For instance, a CD conjugated with an anti-HER2 antibody could enable dual functionality for selective drug delivery and real-time imaging in HER2-positive GBC cells (Oyasiji et al., 2015).

### 5.2 Carbon dot-based targeting of oncogenic signaling pathways in biliary tract cancer

Carbon dots (CDs) and carbon quantum dots (CQDs) have emerged as multifunctional nanoplatforms capable of combining targeted drug delivery, molecular imaging, and stimulus-responsive therapy. Beyond passive accumulation through the enhanced permeability and retention (EPR) effect, surface-functionalized CDs can actively target dysregulated signaling pathways involved in biliary tract cancer (BTC) progression, metastasis, and therapeutic resistance. Although most evidence originates from other solid tumors, these strategies are highly relevant because BTC shares many of the same oncogenic drivers (Yang et al., 2026).

HER2 amplification occurs in approximately 10%–20% of gallbladder cancers, making it an attractive target for receptor-specific nanomedicine. Functionalization of CDs with anti-HER2 antibodies, peptides, or aptamers enables selective drug delivery and fluorescence-guided imaging, potentially improving tumor specificity while reducing systemic toxicity (Cheng, 2024). EGFR, another frequently overexpressed receptor in BTC, promotes proliferation, invasion, angiogenesis, and resistance to apoptosis. EGFR-targeted CD nanocarriers have demonstrated enhanced receptor-mediated internalization and intracellular drug accumulation in epithelial cancer models, suggesting their applicability for EGFR-positive BTC (Pierce and Gomez, 2025). FGFR2 gene fusions, present in approximately 10%–15% of intrahepatic cholangiocarcinomas, represent one of the most clinically actionable molecular alterations. Although selective FGFR inhibitors have improved clinical outcomes, acquired resistance and off-target toxicities remain major limitations. CD-based nanocarriers may enhance intratumoral delivery of FGFR inhibitors while enabling simultaneous fluorescence imaging, thereby improving therapeutic precision (Yang et al., 2026).

Aberrant activation of the PI3K/AKT/mTOR pathway is common in gallbladder carcinoma and regulates cell proliferation, survival, metabolism, epithelial–mesenchymal transition, and chemoresistance. In several preclinical cancer models, CDs have successfully delivered small-molecule inhibitors, siRNA, and microRNA to suppress PI3K/AKT signaling and inhibit tumor growth (Canale et al., 2021). Likewise, dysregulation of the RAS/RAF/MEK/ERK (MAPK) pathway contributes to uncontrolled proliferation and metastatic progression, highlighting the potential of CD-mediated co-delivery systems that combine pathway inhibitors with conventional chemotherapeutics to overcome adaptive resistance (Jaiswar and Dixit, 2026). Active targeting of the tumor microenvironment further enhances the therapeutic potential of CDs. CD44, a marker of cancer stem-like cells and aggressive BTC phenotypes, can be targeted using hyaluronic acid-functionalized CDs to promote receptor-mediated uptake. EpCAM, highly expressed in cholangiocarcinoma, is another promising biomarker for targeted imaging and drug delivery. In addition, folate-conjugated and RGD peptide-functionalized CDs have demonstrated improved tumor selectivity by targeting folate receptors and integrin αvβ3, respectively, both of which are frequently upregulated in hepatobiliary malignancies (Gupta et al., 2023). Despite these advances, pathway-specific CD therapeutics have rarely been evaluated directly in BTC. Most current evidence is extrapolated from hepatocellular, pancreatic, breast, and colorectal cancer models. Future research should therefore focus on validating multifunctional CD platforms in clinically relevant BTC models, integrating molecular targeting with chemotherapy, phototherapy, and real-time fluorescence imaging to address the molecular heterogeneity and therapeutic resistance characteristic of biliary tract malignancies (Chakrabarti et al., 2020). Table 3 summarizes the major oncogenic signaling pathways targeted by CDs and CQDs in preclinical cancer models, the corresponding targeting strategies, therapeutic cargos, experimental models, principal outcomes, and their potential translational relevance to biliary tract cancer.

**TABLE 3:** Cancer-associated signaling pathways targeted by carbon dots and carbon quantum dots in preclinical cancer models with translational relevance to biliary tract cancer.

Molecular pathway/receptor	Biological role in BTC	Carbon dot strategy	Therapeutic cargo	Experimental cancer model	Major findings	Translational relevance to BTC	Ref.
HER2	Cell proliferation, survival	Anti-HER2 antibody-conjugated CDs	DOX, imaging probes	Breast, gastric, liver cancer	Selective uptake and improved cytotoxicity	Applicable to HER2-positive GBC	Cheng (2024)
EGFR	Growth, invasion, metastasis	EGFR-targeted CQDs	DOX, siRNA	Lung, colorectal, HCC	Enhanced receptor-mediated internalization	EGFR overexpressed in BTC	Yang et al. (2026)
FGFR2	Cell growth and angiogenesis	Ligand-functionalized CQDs	FGFR inhibitors	Cholangiocarcinoma	Targeted inhibition with reduced systemic toxicity	Clinically relevant in iCCA	Yang et al. (2026)
PI3K/AKT/mTOR	Cell survival, metabolism	Drug-loaded CDs	PI3K inhibitors	Breast, liver cancer	Reduced proliferation and apoptosis induction	Frequently activated in GBC	Canale et al. (2021)
RAS/RAF/MEK/ERK	Cell-cycle regulation	Dual-drug CQDs	MEK inhibitors + DOX	Pancreatic cancer	Synergistic tumor inhibition	KRAS pathway altered in BTC	Jaiswar and Dixit (2026)
CD44	Cancer stemness, metastasis	Hyaluronic acid-coated CDs	DOX	Breast, liver cancer	Increased cellular uptake	CD44 expressed in BTC	Gupta et al. (2023)
EpCAM	Cell adhesion, tumor progression	Antibody-functionalized CDs	Imaging agents	Cholangiocarcinoma	Improved fluorescence imaging	Highly expressed in CCA	Gupta et al. (2023)
Integrin αvβ3	Angiogenesis	RGD-modified CDs	DOX	Multiple cancers	Enhanced tumor accumulation	Useful for metastatic BTC	Yang et al. (2026)
Folate receptor	Nutrient uptake	Folic acid-functionalized CDs	DOX	Liver cancer	Receptor-specific uptake	Overexpressed in subsets of BTC	Yang et al. (2026)
PD-L1/Immune checkpoint	Immune evasion	Immunomodulatory CQDs (emerging)	Anti-PD-L1 agents	Melanoma, breast cancer	Enhanced immunotherapy response	Future direction for BTC	Yang et al. (2026)

This table highlights that most pathway-targeted CD-based nanotherapeutic systems have been developed in hepatocellular carcinoma and other epithelial malignancies rather than biliary tract cancers. Nevertheless, the considerable overlap in molecular alterations, including HER2 amplification, EGFR overexpression, FGFR2 fusions, PI3K/AKT activation, and CD44-mediated stemness, provides a strong biological rationale for translating these nanoplatforms to BTC. Importantly, future studies should prioritize disease-specific validation rather than assuming equivalent therapeutic responses across tumor types. The establishment of orthotopic gallbladder cancer and cholangiocarcinoma models, together with patient-derived organoids and xenografts, will be essential for confirming the efficacy and safety of these pathway-directed carbon-dot systems before clinical investigation (Chakrabarti et al., 2020). Figure 11 highlight the molecular targets of carbon dots to use in biliary tract cancer.

**FIGURE 11:**
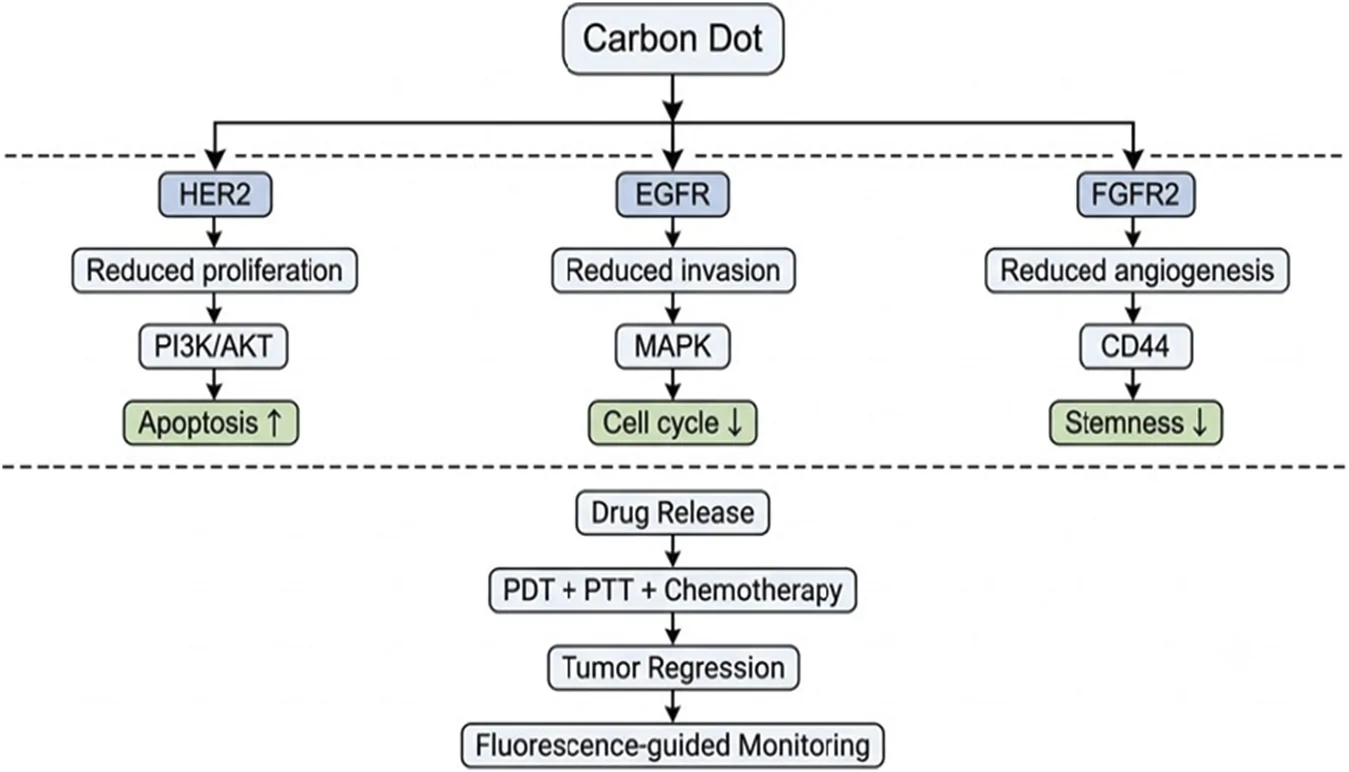
Molecular targets of carbon dots in biliary tract cancer.

## 6 Application of CQDs in biliary tract cancer

To date, direct studies of CQDs in biliary tract cancer (BTC) are limited, reflecting the relative novelty of this field. No clinical trials of CQDs have yet been reported in BTC. However, emerging *in vitro* research provide evidences that CQDs can affect cholangiocarcinoma cells. Notably, Xia et al. (2019) demonstrated that carbon dots synthesized from red bean extract inhibited proliferation of multiple cancer cell lines, including intrahepatic cholangiocarcinoma (HuCCT1) cells (Xia et al., 2019). In that study, CQD treatment reduced cell viability in a time- and dose-dependent manner, impaired cell migration, and induced greater tumor cell death when combined with doxorubicin than either agent alone. This suggests that CQDs can function both as cytotoxic agents and as adjuvants to chemotherapy in BTC cells. The mechanism may involve CQD induced stress pathways or enhanced drug uptake, but specifics remain under investigation (Xia et al., 2019).

Aside from this, most insights must be extrapolated from analogous models. In hepatobiliary research, the potential of CQDs can be inferred from studies in hepatocellular carcinoma (HCC) and pancreatic cancer, which share metabolic and stromal features with cholangiocarcinoma. For imaging, CQD-based contrast has been used to visualize liver tumors and biliary structures *in vivo* (rodent models) (Bayda et al., 2021). For targeted delivery, nanocarriers functionalized with peptides that bind cholangiocarcinoma markers (e.g., EGFR, FGFR2 fusions) could theoretically be grafted onto CQDs. In principle, CQDs bearing an anti-EGFR affibody or an antibody fragment might home to EGFR-overexpressing BTC cells, paralleling strategies explored in other cancers (Dana et al., 2025). Similarly, PDT-based approaches targeting BTC are of interest: CQDs that generate singlet oxygen under NIR illumination could be applied intrahepatically or endoscopically to ablate cholangiocarcinoma foci, particularly for unresectable perihilar lesions (Hendriquez et al., 2022). Several indirect pieces of evidence reinforce this potential. The general merits of nanoparticle therapy in BTC have been reviewed: for example, albumin-bound paclitaxel (nab-PTX) and liposomal irinotecan are under clinical evaluation for cholangiocarcinoma, indicating openness to nanoformulations. Moreover, gold- and lipid-based nanoparticles targeting cholangiocarcinoma (via folate or HER2) have shown promising results in cell lines (Dana et al., 2025).

Diagnostics is another emerging application. Early detection of BTC is notoriously difficult due to deep location and lack of symptoms. Fluorescent CQDs might serve as imaging agents during endoscopy or surgery (Naghavi, 2015). For example, fluorescent cholangiography (using indocyanine green) is already practiced; a brighter, targeted CQD probe could improve visualization of small tumors or bile duct margins. CQDs conjugated to tumor markers (such as mucin-1 or glypican-3) could enable tumor-specific fluorescence imaging of resected specimens or *in vivo* bile ducts (D’Acapito et al., 2023).

In general, although *in vivo* and clinical data on CQDs in BTC are still lacking, available evidence and analogies suggest multiple avenues for their use. CQDs could be engineered for targeted drug delivery or theranostic imaging in cholangiocarcinoma, potentially enhancing the efficacy of chemotherapy or enabling image-guided interventions. The promising *in vitro* cytotoxicity of CQDs against cholangiocarcinoma cells indicates that further preclinical investigation is warranted. Future studies should explore CQD formulations in relevant BTC animal models (e.g., orthotopic xenografts of cholangiocarcinoma), combining the known strengths of CQDs (fluorescence, drug loading) with BTC-specific targeting (e.g., bile duct tropism, receptor expression profiles) (Dana et al., 2025).

## 7 Toxicity and biocompatibility of CQDs

Safety is paramount for any nanomaterial, particularly for clinical translation. Fortunately, CQDs generally exhibit far lower toxicity than heavy-metal quantum dots. Many studies report that CQDs are non-toxic at functional doses: for instance, it was found that a variety of biomolecule derived CQDs showed no cytotoxicity up to 0.4 mg/mL in cell culture (Zhao et al., 2008). Similarly, it was also noted that CQDs from natural sources are prized for their biocompatibility and non-toxicity (Wang and Hu, 2014). *In vivo* rodent studies often corroborate that animals tolerate intravenous CQD doses on the order of tens of mg/kg without obvious clinical toxicity. One comparative study in zebrafish embryos found ∼95% survival at 100 ppm of green-synthesized CQDs (only 5% embryo mortality), whereas metallic Cd-based QDs cause near-complete lethality at comparable doses (Liu et al., 2020). This suggests a substantially wider safety margin for CQDs.

In a recent study, the biocompatibility of the carbon dots (CDs) was systematically evaluated using two tumor cell lines (HepG2 and HeLa) and two normal cell lines (7,702 and 293T) as shown in Figure 10. Cytotoxicity assays showed that even at a high concentration of 400 μg/mL, cell viability remained above 86% after 24 h and above 70% after 48 h, with no significant differences among the four cell types (P > 0.05), indicating minimal toxicity. Flow cytometry analysis of 7,702 cells further confirmed negligible apoptosis, with early and late apoptosis rates remaining comparable to control cells after 24 and 48 h incubation. These results collectively demonstrate that the CDs possess excellent biocompatibility, are non-toxic to both normal and tumor cells, and are therefore suitable as carriers for targeted anticancer drug delivery and therapy (Wang S, 2020). Figure 12 shows the results for different concentration of Cds on different cell lines.

**FIGURE 12:**
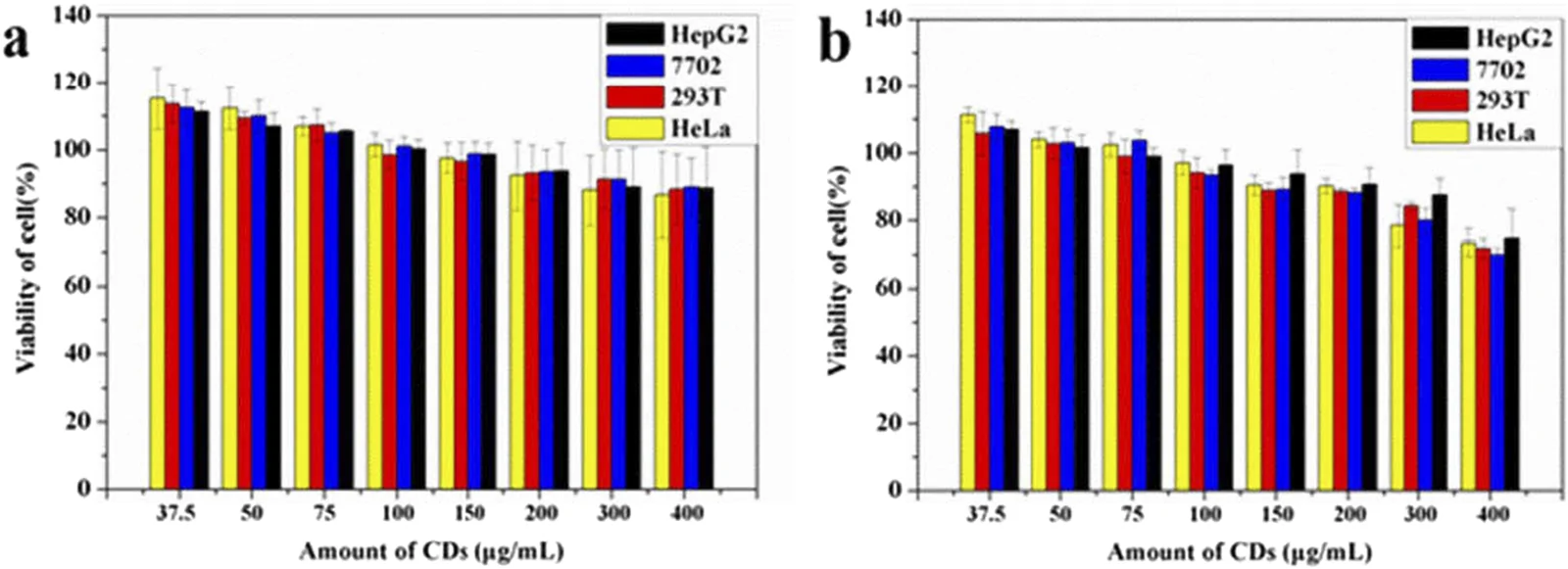
Viability of HepG2, HeLa, 7,702 and 293 T cells after incubating with CDs for 24 h **(a)** and 48 h **(b)**. Reprint with permission from (Shicai Wang), (Enhanced-fluorescent imaging and targeted therapy of liver cancer using highly luminescent carbon dots-conjugated foliate); published by (Elsevier), (2020) (Wang S, 2020).

Nevertheless, CQD biocompatibility is not guaranteed and depends on synthesis. Key factors influencing toxicity include precursor materials, particle size, surface charge, and residual reagents (Atchudan et al., 2022) (Zhu et al., 2019). Some precursors or dopants can introduce harmful elements, and synthesis by-products (acids, organic solvents) must be carefully removed. For example, CQDs prepared with strong acid oxidation (graphite + nitric acid) require thorough purification to avoid nitrate contamination. Surface charge also matters: highly positive CQDs can cause cell membrane damage, whereas neutral or PEGylated CQDs are generally more benign (Smith et al., 2008). In biodistribution, CQDs tend to accumulate first in the reticuloendothelial system (liver, spleen) as part of normal NP clearance, but their small size often leads to partial renal excretion. Long-term accumulation studies are scarce; however, one study showed that CQDs cleared from major organs within weeks, with no histopathologic damage observed in liver or kidney tissue at low medium doses (Liao et al., 2021). Despite the low acute toxicity seen in initial studies, systematic toxicity profiling is needed. It is observed that the lethal dose and chronic effects *in vivo* remain largely unquantified (Huang et al., 2013).

Factors such as oxidative stress generation, immunogenicity, and interactions with blood components are understudied. It is also known from broader nanotoxicology that nanoparticles can have unforeseen effects (e.g., protein corona formation, endothelial activation) (Huang et al., 2013). Regulatory guidelines for nanomaterials are evolving, and CQDs fall into a gray zone. On one hand, their carbon composition is generally regarded as safe (e.g., carbon nanoparticles in other contexts have passed safety trials), but on the other hand CQDs are engineered materials with unique properties requiring new assessment. In particular, biodot CQDs derived from food or waste sources may contain complex organic residues requiring careful characterization (Ma, 2024).

In general, current evidence suggests CQDs are relatively biocompatible as they exhibit negligible cytotoxicity at practical doses, and animal studies report high survival even with high CQD concentrations. However, generalizing this safety requires caution. Each CQD formulation must be evaluated for impurities, immune reactions, and long-term effects. Importantly, for BTC applications one must consider biliary and hepatic effects specifically. The liver, which often filters nanoparticles, could be particularly exposed. Preliminary data indicate CQDs are not inherently hepatotoxic, but dedicated studies of cholangiocyte or hepatocyte function in the presence of CQDs would be prudent. Overall, toxicity data are encouraging but incomplete: rigorous pharmacokinetics, repeat-dose toxicity, and genotoxicity studies are still needed before clinical use (Yao et al., 2019).

## 8 Challenges and limitations for CDs and CQDs

While CQDs hold promise, several challenges must be overcome to translate them to the clinic, especially in a difficult context like BTC. Technical hurdles include standardizing CQD synthesis. Current protocols (especially “green” or bottom-up methods) often yield heterogeneous products. As the literature notes, parameters such as precursor temperature, reaction time, pH, and dopant ratios critically affect CQD size, surface chemistry, and hence optical and biological properties (Yang et al., 2015). Without rigorous control, batch-to-batch variability could impair reproducibility and safety. Furthermore, many promising CQD formulations are reported only at lab scale. Scaling up production (e.g., gram or kilogram quantities) while maintaining quality is non-trivial. There is currently “no thorough manual” or industrial roadmap for large-scale CQD manufacture. Addressing this will require collaboration between chemists and process engineers, possibly adapting continuous-flow or microwave-assisted synthesis for uniformity (Aniogo et al., 2019).

Pharmacokinetic and delivery barriers also remain. Although small, CQDs still face opsonization and clearance by macrophages. Achieving selective delivery to deep-seated bile duct tumors will be challenging: EPR in cholangiocarcinoma is modest due to hypovascular stroma (Dana et al., 2025). Active targeting ligands may enhance specificity, but their clinical efficacy has historically been mixed. Additionally, CQDs must penetrate the dense fibrotic tissue of BTC. Strategies like nanoparticle-embedded hydrogels or microneedle delivery (for peri-hilar tumors) could be explored, but add complexity. Phototherapy applications face the limitation that light penetration in liver is limited; only intraoperative or interstitial illumination might be effective for deep tumors (Manzari et al., 2021) (Bhattacharya et al., 2023). Moreover, nanomedicine trials in oncology have historically faced difficulties in patient accrual and demonstrating benefit above standard therapy. In BTC, where effective therapies are scant, there may be opportunity for early-phase studies, but outcomes such as survival would need long follow-up (Ma, 2024). Finally, biological complexity of BTC imposes challenges. BTC tumors are heterogeneous (distinct subtypes like intra-vs. extra-hepatic have different genetics) and any nanotherapy may only suit a subset. Identifying the right patient population (e.g., FGFR2-fusion iCCA vs. others) will be key. Moreover, the tumor microenvironment in BTC is immunosuppressive and fibrotic; nanocarriers may need to be combined with stroma-modulating agents or immunotherapies. In summary, the path from bench to bedside will require overcoming synthetic, biological, and regulatory complexities (Banales et al., 2020) (Kratz et al., 2024).

### 8.1 Current limitations, conflicting evidence, and barriers to clinical translation of carbon dots in biliary tract cancer

Despite remarkable progress in the development of carbon dots (CDs) and carbon quantum dots (CQDs) for cancer theranostics, several scientific and translational challenges continue to hinder their progression toward clinical application, particularly in biliary tract cancers (BTCs). While numerous preclinical investigations have reported promising results in terms of drug delivery efficiency, fluorescence imaging, phototherapy, and biosafety, the available evidence remains fragmented, with considerable variability in nanoparticle synthesis, physicochemical characterization, experimental design, and biological evaluation. Consequently, direct comparison among studies is often difficult, limiting the reproducibility and generalizability of published findings (Bartkowski et al., 2024). One of the major limitations is the absence of standardized synthesis protocols. Carbon dots produced through hydrothermal, microwave-assisted, solvothermal, electrochemical, or laser-ablation methods frequently exhibit substantial differences in particle size, crystallinity, surface chemistry, quantum yield, and functional-group density. Even minor variations in precursor composition, reaction temperature, synthesis duration, or purification procedures may significantly alter their optical properties, drug-loading efficiency, biodistribution, and biological activity. Such batch-to-batch variability represents a considerable obstacle for regulatory approval and large-scale manufacturing because reproducible physicochemical characteristics are fundamental requirements for pharmaceutical nanomaterials (Salvi et al., 2024).

Another important challenge concerns the limited understanding of nanoparticle pharmacokinetics and biodistribution. Although CDs are generally considered highly biocompatible because of their ultrasmall dimensions and hydrophilic surface chemistry, comprehensive studies evaluating absorption, systemic distribution, metabolism, biliary and renal clearance, and long-term organ retention remain scarce. Since biliary tract cancers develop within organs responsible for nanoparticle metabolism and excretion, understanding the interaction of CDs with hepatocytes, cholangiocytes, Kupffer cells, and the reticuloendothelial system is particularly important. Small changes in nanoparticle surface charge or functionalization may substantially alter circulation time, hepatic uptake, and clearance kinetics, ultimately influencing therapeutic efficacy and safety (Bartkowski et al., 2024). A further complexity arises from the formation of the protein corona, whereby plasma proteins rapidly adsorb onto the nanoparticle surface immediately after systemic administration. This dynamic biological coating may mask targeting ligands, modify nanoparticle size and surface charge, alter cellular uptake mechanisms, activate complement pathways, and significantly influence biodistribution. Consequently, the biological identity of CDs *in vivo* may differ substantially from their physicochemical characteristics measured under laboratory conditions. Recent investigations have emphasized that protein corona formation should be routinely evaluated during preclinical development because it directly affects targeting specificity, immune recognition, and therapeutic performance (Morbidelli et al., 2024).

Although most published studies report low acute toxicity, long-term biosafety remains insufficiently characterized. Current investigations predominantly evaluate short-term cell viability or limited-duration animal studies, whereas chronic toxicity, repeated-dose exposure, reproductive toxicity, genotoxicity, immunotoxicity, and carcinogenic potential have rarely been systematically investigated. Furthermore, differences in precursor materials, heteroatom doping, residual synthesis reagents, and purification efficiency may produce substantial variability in toxicological profiles among apparently similar CD formulations. Therefore, the favorable safety profile reported for one formulation cannot be generalized to all carbon-dot systems without rigorous formulation-specific evaluation (Bhattacharya et al., 2024).

An additional limitation is the scarcity of disease-specific experimental models. Most available evidence supporting CD-mediated cancer therapy has been generated using hepatocellular carcinoma, breast cancer, colorectal cancer, pancreatic cancer, or cervical cancer models, whereas direct investigations involving gallbladder carcinoma and cholangiocarcinoma remain extremely limited. Although these malignancies share several molecular alterations, including activation of EGFR, HER2, PI3K/AKT/mTOR, and MAPK signaling pathways, their tumor microenvironment differs substantially. Biliary tract cancers are characterized by extensive desmoplastic fibrosis, poor vascularization, hypoxia, dense extracellular matrix deposition, and profound immune suppression, all of which may restrict nanoparticle penetration and therapeutic delivery. Consequently, therapeutic responses observed in other solid tumors cannot be directly extrapolated to BTC without validation in orthotopic animal models, genetically engineered mouse models, and patient-derived organoids (Bartkowski et al., 2024). Manufacturing and regulatory considerations also remain major barriers to clinical translation. Before human application, CD formulations must be produced under Good Manufacturing Practice (GMP) conditions with strict control over particle size distribution, purity, sterility, endotoxin content, stability, and reproducibility. Regulatory agencies additionally require comprehensive pharmacokinetic evaluation, toxicological assessment, immunogenicity testing, and validated analytical methods before first-in-human studies can be initiated. Because no internationally standardized manufacturing framework currently exists for carbon dots, regulatory approval pathways remain less defined than for conventional pharmaceutical products. This lack of harmonization continues to delay industrial development and commercialization despite encouraging laboratory-scale results. Finally, future investigations should shift from demonstrating proof-of-concept therapeutic efficacy toward establishing clinically relevant translational evidence (Salvi et al., 2024).

### 8.2 Future perspectives and clinical translation

Despite these challenges, the field is rapidly advancing and innovations are on the horizon. Future research directions include optimizing CQD design for deeper tissue imaging and therapy. One limitation noted in the literature is the lack of NIR-II–emitting CQDs (1,000–1700 nm) for deep-tissue imaging (Dirheimer et al., 2022). Developing red/NIR-emissive CQDs (via controlled doping or novel precursors) would greatly enhance utility in BTC, enabling intraoperative fluorescence at 1,000–1,300 nm where liver tissue autofluorescence is minimal. Similarly, enhancing photothermal conversion efficiency by combining CQDs with plasmonic or semiconductor nanomaterials could yield potent photothermal agents for ablation of resistant tumor regions (Yang et al., 2015).

Machine learning is an emerging tool that may accelerate CQD design. Recent reports suggest AI-guided optimization can predict CQD precursors and synthesis conditions that yield desired emission properties (Singh et al., 2020). Applying such approaches could rapidly produce libraries of CQDs tailored for specific tasks (e.g., targeting mitochondrial membranes, or crossing the blood–tumor barrier). One exciting avenue is the development of CQDs capable of crossing the blood–brain barrier for CNS metastases; while not directly BTC-related, it illustrates the potential of engineered CQDs for challenging delivery tasks (Zhang et al., 2021) (Lu, 2016). In a recent research work, Chen et al. (2023) developed a machine learning (ML) assisted strategy to control the synthesis of multicolor carbon dots (CDs) with tunable optical properties as shown in Figure 13. By conducting 270 synthesis experiments under varied conditions, they trained and optimized several ML models, identifying the Random Forest algorithm as the most accurate for predicting emission wavelength, quantum yield, and Stokes shift. The model revealed that solvent type predominantly determines emission wavelength, precursor ratio affects quantum yield, and precursor type influences Stokes shift. These insights enabled the rational design of stable blue red emissive CDs, later applied in multicolour information encryption. This study demonstrates that ML can effectively uncover complex synthesis property relationships and guide the data driven design of the highest performance luminescent carbon nanomaterials (Chen et al., 2023).

**FIGURE 13:**
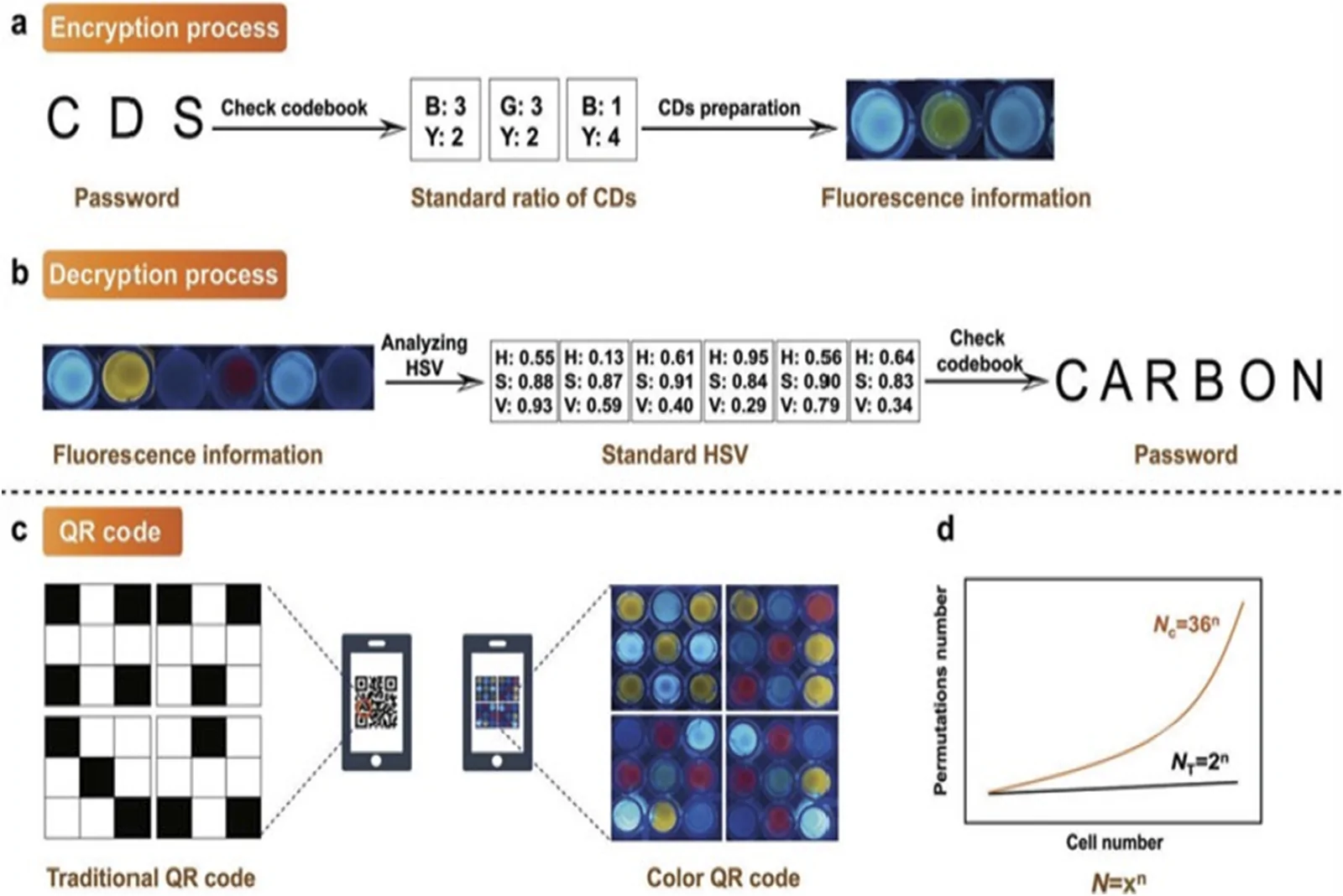
Informative transmission of multicolour carbon dots by machine learning (Chen et al., 2023). Reprint with permission from (Jiao Chen), (Controlled Synthesis of Multicolor Carbon Dots Assisted by Machine Learning); published by (wiley), (2022) (Chen et al., 2023).

In terms of BTC-specific translation, combination therapies integrating CQDs with existing treatments are promising. For instance, CQDs could be loaded with gemcitabine or FGFR inhibitors and tested in combination with immunotherapy (checkpoint inhibitors), potentially converting the immunologically cold BTC microenvironment into a more responsive one (Soumya et al., 2023). In the Kras- or IDH1-mutant BTC mouse models currently used in preclinical studies, CQD-mediated delivery of targeted small molecules could be evaluated. The KIWI-CDs example shows biowaste-derived CQDs can be safe and multifunctional. Similar eco-friendly approaches could be adopted for large-scale production of medical grade CQDs (O’Hagan and Heyer, 2011).

Clinically, the first CQD trials in BTC will likely be small-scale safety studies, possibly in conjunction with surgical exploration or ablation. For example, a possible trial could administer CQDs intravenously prior to surgery, then use fluorescence laparoscopy to visualize occult metastases (sentinel lymph nodes, small nodules) a strategy analogous to sentinel node mapping (Sarkar et al., 2024). Another conceivable application is endoscopic delivery: fluorescent CQDs sprayed or injected into the biliary tree during ERCP to guide biopsy or phototherapy. In therapy, CQD–drug conjugates might enter Phase I studies if they show compelling efficacy in animal models with manageable safety. Importantly, regulatory agencies may allow accelerated pathways for BTC indications due to the unmet need (Bodele et al., 2025). Commercial prospects are growing as nanomedicine penetrates the oncology market. Several companies now offer CQDs for research use; a few biotechnology firms are exploring CQD products though mostly for imaging (Naik et al., 2022). In the context of BTC, collaboration between nanotech firms and oncology centers could catalyze progress. Regulatory agencies including the U.S. Food and Drug Administration (FDA) and the European Medicines Agency (EMA) increasingly recommend a Quality-by-Design (QbD) approach for nanomedicine development, emphasizing comprehensive characterization of critical quality attributes, reproducible manufacturing processes, pharmacokinetics, biodistribution, immunotoxicity, and long-term biosafety. In addition, compliance with International Council for Harmonisation (ICH) quality guidelines and Good Laboratory Practice (GLP) toxicological evaluation is expected before initiation of first-in-human clinical trials. For multifunctional theranostic CQDs integrating imaging, drug delivery, and phototherapy within a single nanoplatform, regulatory approval pathways may become even more complex because each functional component requires independent demonstration of safety, efficacy, and manufacturing reproducibility. Investments in GMP-grade CQD manufacturing and regulatory consulting will be needed (Dwivedi et al., 2015). Future clinical translation should therefore focus on developing disease-specific CQDs capable of selectively targeting molecular biomarkers associated with biliary tract cancers, including HER2, EGFR, FGFR2, EpCAM, and CD44, while simultaneously integrating multimodal imaging, controlled drug release, and phototherapeutic functionality. The combination of artificial intelligence-assisted nanomaterial design, patient-derived tumor models, and precision oncology approaches may further accelerate optimization of CQD formulations for individualized treatment. Collectively, overcoming current manufacturing, pharmacological, regulatory, and toxicological limitations will be essential for translating CQD-based nanotheranostic platforms from promising experimental systems into clinically applicable precision medicines for biliary tract cancer. Encouragingly, precedent for carbon nanomaterials in the clinic is emerging: while not cancer-related, a carbon nanoparticle (C-dots) was approved for lymphatic mapping, demonstrating that such materials can meet regulatory standards (Lisik and Krokosz, 2021). The clinical implementation of CD/CQD-based therapeutics for BTC requires overcoming several critical challenges, including limited tumor-specific validation, incomplete pharmacokinetic and toxicological characterization, manufacturing scalability, reproducibility, and regulatory standardization. These key translational barriers are summarized in Table 4.

**TABLE 4:** Current barriers limiting the clinical translation of carbon dots for biliary tract cancer.

Challenge	Current limitation	Clinical implication	Future direction	Ref.
Synthesis reproducibility	Batch-to-batch variability	Poor reproducibility	Standardized GMP synthesis	Bhattacharya et al. (2024)
Particle characterization	Variable size and surface chemistry	Difficult regulatory approval	International characterization guidelines	ChenLiu et al. (2025)
Pharmacokinetics	Limited biodistribution data	Unknown long-term safety	Comprehensive ADME studies	Ying et al. (2025)
Protein corona	Alters targeting efficiency	Reduced therapeutic specificity	Surface engineering and corona optimization	Canale et al. (2021)
Long-term toxicity	Few chronic studies	Uncertain clinical safety	Repeat-dose and genotoxicity studies	Gupta et al. (2023)
BTC-specific evidence	Very limited animal models	Weak translational evidence	Orthotopic GBC and CCA models	Gupta et al. (2023)
Tumor microenvironment	Dense fibrotic stroma	Poor nanoparticle penetration	Active targeting and stroma-modulating strategies	Yang et al. (2026)
Regulatory approval	No standardized pathway	Delayed commercialization	GMP manufacturing and harmonized regulations	Dwivedi et al. (2015), (Canale et al. (2021)

## 9 Conclusion

Biliary tract cancers (BTC), which includes cholangiocarcinoma and gallbladder cancer, still remains the most therapeutically challenging malignancies due to their profound molecular heterogeneity, desmoplastic stroma, poor vascularity, late clinical approaches, and limited responsiveness to customary chemotherapy. The onset of nanomaterials of carbon based, particularly carbon quantum dots (CQDs) offer an excellent opportunity to overcome these longstanding barriers. This review demonstrates, the structural evolution from amorphous carbon dots (CDs) to highly crystalline CQDs has yielded nanoplatforms with superior photophysical properties, enhanced quantum yield, tunable emission extending into the NIR region, improved rate of charge transfer, and greater suitability for multimodal cancer theranostics. CQDs have a unique combination of advantages as they have very small size, have facile functionalization, providing great biocompatibility, and inherent fluorescence that collectively enable targeted drug delivery, real-time imaging, photodynamic therapy, photothermal ablation, and synergistic nano-chemo treatments. Even though direct research on CQDs in biliary tract cancers remains limited, emerging evidence from cholangiocarcinoma cell lines, hepatobiliary tumor models, and related gastrointestinal malignancies provides compelling evidences for their applicability. Previous studies indicate that CQDs can inhibit tumor proliferation, enhance chemotherapeutic potency, can also improve intracellular drug accumulation, and deliver image guided therapy with minimal systemic toxicity. Despite these promising attributes, several challenges must be addressed before CQDs can enter translational or clinical pipelines for BTC. For examples, standardization of synthesis method, rigorous toxicological testing, strong biodistribution profiling, and scalable production are still remains underdeveloped. Furthermore, BTC specific targeting strategies such as HER2, FGFR2, EpCAM, or CD44-directed CQDs, and clinically relevant *in vivo* BTC models must be systematically developed. Integrating CQDs with current and emerging treatment modalities could include immunotherapy, molecular targeted agents, and minimally invasive image-guided surgery, representing a particularly promising direction. In general, CQDs exemplify a next-generation nanotheranostic platform with the potential to fundamentally reshape how biliary tract cancers could be diagnosed, monitored, and treated. Continued interdisciplinary research will bridge with nanotechnology, hepatobiliary oncology, and translational pharmacology to unlock the full clinical potential of CQDs and advancing them toward first in human applications for biliary tract cancer.
